# Quantum dot molecular beacons achieve sub-10 pM CRISPR-Cas detection in field-ready assays

**DOI:** 10.1038/s41598-025-09434-9

**Published:** 2025-07-31

**Authors:** Drew P. Lysne, Michael H. Stewart, Kimihiro Susumu, Tomasz A. Leski, David A. Stenger, Igor L. Medintz, Sebastián A. Díaz, Christopher M. Green

**Affiliations:** 1 National Research Council, 500 Fifth St NW, Washington, DC 20001 USA; 2 U.S. Naval Research Laboratory, Center for Bio/Molecular Science and Engineering, Code 6900, Washington, DC 20375 USA; 3 U.S. Naval Research Laboratory, Optical Sciences Division, Code 5600, Washington, DC 20375 USA

**Keywords:** CRISPR/Cas, Molecular beacons, Quantum dots, Förster resonance energy transfer, Cell-phone detection, Nanobiotechnology, Assay systems, Biophotonics

## Abstract

CRISPR-Cas systems have revolutionized molecular diagnostics through their specificity and programmability, yet their broad adoption is hindered by the reliance on expensive and complex instrumentation. Here, we present an optimized quantum dot (QD) molecular beacon (QD-MB) platform that integrates Förster resonance energy transfer (FRET)-based detection with CRISPR-Cas functionality, achieving sub-picomolar sensitivity without the need for target amplification. By systematically tuning components, including His-tag modifications for improved QD conjugation, nucleic acid hairpin structures for enhanced enzyme interaction, and QD surface passivation strategies, we demonstrate a two-order-of-magnitude improvement in detection sensitivity. Using LwaCas13a and RNA targets, the limit of detection (LOD) decreased to under 1 pM with plate-reader-based fluorescence measurements and below 10 pM with a lamp-and-smartphone setup, establishing the feasibility of portable, field-ready applications. This work highlights the transformative potential of QD-MBs in biosensing and sets a foundation for further advances in CRISPR-based diagnostics and nanotechnology-enabled sensing platforms.

**Supplementary Information:**

The online version contains supplementary material available at 10.1038/s41598-025-09434-9.

## Introduction

Clustered Regularly Interspaced Short Palindromic Repeats, or more commonly known as CRISPR, are 20–50 nucleotide long DNA sequences found in the genomes of archaea and bacteria^1,2^. These sequences are used in archaeal and bacterial adaptive immune responses to invading bacteriophages, coding short snippets of viral genomes for recognition of viral presence^3–5^. The CRISPR DNA fragments are then transcribed into guide RNA (gRNA), which binds with CRISPR-associated (Cas) proteins and undergoes a search for nucleic acids present within the cell^6^. Upon identification of viral material complementary to gRNA, Cas enzymes induce site-specific cleavage of the invading nucleic acids. A plethora of Cas proteins have been discovered, with the majority of interest directed at class 2 Cas proteins, which possess the comprehensive functionality to recognize and cleave invasion strands without assistance from other proteins^7–10^. Among the class 2 Cas proteins, types II (e.g., Cas9), V (e.g., Cpf1/Cas12), and VI (e.g., C2c2/Cas13) are notable. Types V and VI are additionally distinct among Class 2 Cas enzymes because of their ability to cleave nucleic acids nonspecifically upon activation by a target^9,11,12^. Once these Cas enzymes are activated by the target that complements the internal gRNA, they realize multiple turnovers of non-specific DNA or RNA cleavage, creating an optimal signal transduction and amplification mechanism.

The Zhang Lab at MIT helped pioneer CRISPR diagnostic strategies by leveraging Cas enzyme trans-cleavage to create sensitive and specific nucleic acid detection assays. Their method, termed specific high-sensitivity enzymatic reporter unlocking (SHERLOCK) was developed using Cas13a ortholog LwCas13a^13^. Paired with recombinase polymerase amplification, they achieved attomolar (aM) detection of the Zika virus in a fluorescence detection assay. Similarly, the Doudna Lab at UC Berkeley developed a DNA endonuclease-targeted CRISPR trans reporter (DETECTR) that utilizes Cas12a to transduce and amplify the detection of a DNA target through non-specific cleavage of a DNA-based molecular beacon^11,14^. Numerous approaches with distinct Cas enzymes and modified reporting strategies have since been reported, ranging from colorimetric and electrochemical readout to magnetic bead-based approaches and nanodroplet manipulation^15^. Despite these alternative approaches, fluorophore/quencher molecular beacons (FQ-MB) used for microplate fluorescence measurements remain the most common readout strategy for CRISPR/Cas biosensing because of their simplicity and ease of use.

Although FQ molecular beacons remain the predominant method in CRISPR diagnostics, this method requires sensitive plate readers and/or optical bandpass filters for both excitation and readout. As an alternative, we have developed a quantum dot (QD)-based MB that provides ratiometric Förster resonance energy transfer (FRET)-based detection^16^. As fluorescent donors, QDs have ideal optical properties (high brightness, broad excitation, narrow emission, photostability, and large surface/volume ratio) and sufficient surface area to bind multiple acceptors, significantly increasing FRET efficiencies^17–20^. Enzymatic cleavage of the substrate (e.g., peptides and nucleic acids) releases fluorescence acceptors from the QD, leading to reduced FRET and large ratiometric spectral changes, which can yield quantitative measurements of target biomarkers. In a recent study, we demonstrated the effectiveness of QD-MB when paired with CRISPR/Cas detection strategies for both DNA and RNA, even allowing multiplexing^16^. For Cas13-based RNA detection without target amplification, a limit of detection (LOD) of 100 picomolar (pM) was achieved, rivaling the performance of FQ-MBs while overcoming many of the limitations associated with fluorophore-based readout. Furthermore, the advantages of QD-MB signaling, particularly the QD’s broad excitation and large emission offset, allowed for the implementation of a low-tech lamp-and-phone camera read-out approach that reported 5 nanomolar (nM) of the target.

The integration of QD-MBs with CRISPR-Cas has improved the readout strategy for nucleic acid detection; however, the optimization of their performance remains necessary. To improve the functionality of QD-MB detection strategies, we tuned various components, including histidine tags (His-tags) that anchor fluorophores to the QD surface; the length, composition, and density of nucleic acid hairpins; and the QD surface coating. The study of these variables provided greater insight into the enzyme-MB interaction, resulting in a two-order-of-magnitude increase in the sensitivity of the QD-MB CRISPR-Cas systems. Specifically, while maintaining the focus on non-amplified assays, the LOD decreased from 100 pM to under 1 pM within a 60 min time window, and even further with longer periods, using a high-throughput plate reader. Additionally, using a low-tech lamp and phone camera, we detected target concentrations below 10 pM. Overall, our results make CRISPR-Cas detection a more viable field-forward sensing technique, and offer guidelines for other QD-based nuclease FRET reporters.

## Results and discussion

### QD-MB assay design and mechanism

**Fig. 1:**
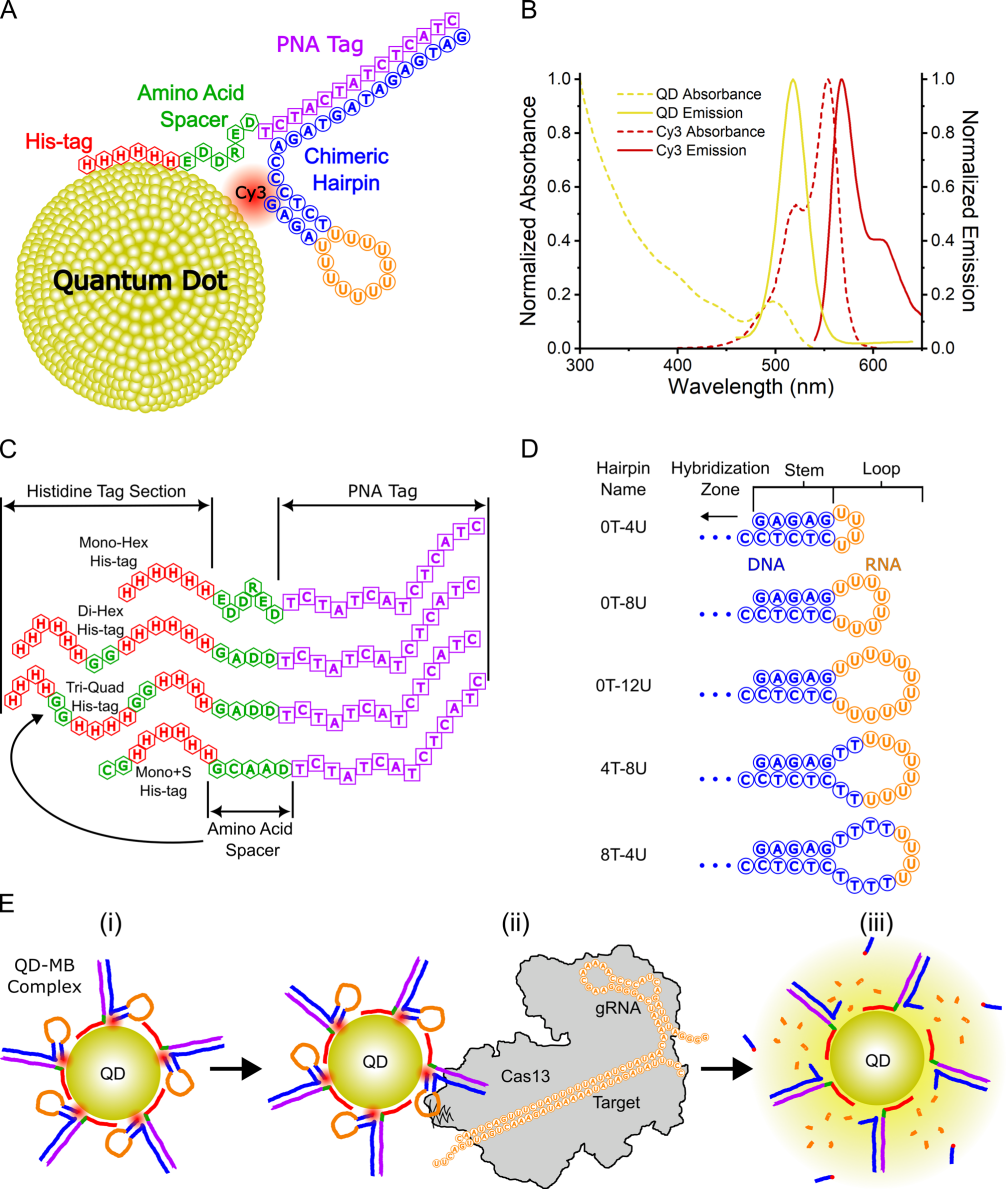
Experimental system schematics and optical properties (**A**) A schematic of a quantum dot molecular beacon (QD-MB) illustrated with one hairpin complex (peptide-PNA: DNA/RNA hairpin). (**B**) Normalized extinction coefficient and emission spectra of both the Cy3 fluorophore and QD. (**C**) Detailed schematic of the peptide-PNA strands including His-tag distinctions. (**D**) Detailed schematics of the five tested hairpins and their compositions. (**E**) (**i**) Schematic of the assembled QD-MB with five hairpin complexes. (**ii**) Activated CRISPR-Cas nucleases cleave the RNA hairpins. (**iii**) Digested hairpins release the short DNA-Cy3 complement to shift the emission spectra.

The principal results focused on understanding and optimizing the variables of the QD-MB system, which has previously demonstrated pM and nM detection levels without amplification using spectrophotometers and cellphone cameras, respectively. As the foundational design principles were outlined in Green et al.^16,21^ a brief description of the function mechanism is provided along with the schematics in Fig. 1. In this system, a dye-labeled RNA or DNA hairpin (RHP/DHP) is immobilized on the surface of a fluorescent semiconductor QD through hybridization with a chimeric peptide/peptide nucleic acid (PNA) that includes a His-tag for binding to the ZnS surface of the QD, as shown in Fig. 1A. Prior to cleavage, the fluorescence of the QD is quenched by the surrounding dyes via FRET. Upon capturing a target nucleic acid and subsequent activation of the Cas nuclease activity, the hairpin is cleaved, releasing the dyes from the complex; as a result, QD fluorescence is restored, while the sensitized dye emission is effectively eliminated.

Building off prior work, we hypothesized that optimization of several components of the QD-MB would improve performance: (1) the FRET pair, composed of the QD donor and any fluorophore acceptors assembled on the surface of the QD; (2) the peptide-PNA, responsible for conjugation of the dye-labeled substrate to the QD surface via a His-tag; (3) the substrate, a dye-labeled nucleic acid hairpin that is readily cleaved by active Cas nucleases to signal the presence of the target; and (4) passivation agents to minimize unintended Cas conjugation to the QDs via His-tags present on the Cas. In this study, we focused on optimizing peptide-PNA, substrate, and passivation agents, as these components directly influence the efficiency of Cas enzyme activity and substrate cleavage. This work used a high-throughput TECAN microplate reader to excite the QDs and collect ratiometric emission data of the QD donor and Cy3 acceptor. Detailed experimental descriptions of all assays are provided in the Materials and Methods section.

The FRET pair, which is critical for signal readout, likely operates independently of the cleavage mechanism and does not affect the enzyme activity. Therefore, changes in the FRET pair primarily influence the signal detection sensitivity rather than the core functional aspects of the QD-MB system. As such, we have included a discussion, but the experimental optimization was limited. The chosen FRET pair is composed of CdSe/CdS/ZnS QDs (4.1 ± 0.5 nm diameter, 525 nm emission peak), coated with a short zwitterionic surface ligand (CL4), as the donor and Cy3 organic fluorophores as the acceptor^22^. This FRET pair has a Förster radius (*R*_0_) of 5.5 nm assuming a dynamic averaging of κ^2^. Although many other variations of QD/dye FRET pairs have been demonstrated, we found that this FRET pair remains a practical and effective choice. The smaller QDs maximize colloidal stability, permit small donor-acceptor distances (*r*_DA_), and minimize excess surface area capable of capturing Cas enzymes (*vide infra*). Similarly, the Cy3 dye is bright and readily available commercially conjugated to DNA and RNA, and although its fluorescence is susceptible to temperature variations, as reported previously^21^, the assay is generally performed at or near room temperature, minimizing fluctuations due to temperature throughout experiments. Additionally, the offset in the donor and acceptor emission spectra allows the simple separation of red and green channels in RGB phone-camera images to measure relative photoluminescence (PL) ratios. While the design is amenable to other variations, we did not find a justification to modify the chosen FRET pair and instead focused on the components affecting the sensitivity and stability of the system.

### Engineering Peptide-PNA conjugates for enhanced QD binding

For QD-based molecular beacons, peptide-PNA was developed as a self-assembly method for conjugating nucleic acids onto surfaces that are compatible with His-tag binding. For ZnS-coated QDs, peptide-PNAs containing His-tags offer reduced proximity to the surface compared to avidin-biotin methods, and are more robust and cost-effective than hybrid peptide-DNA approaches^23^. His-tags, short peptides typically consisting of six contiguous histidines, were originally used as protein tags for purification but are now widely utilized for bioconjugation to nanoparticles. His-tags coordinate with Zn^2+^ ions on the QD surface with a relatively high affinity (*K*_D_ ~ 1 nM) through multi-valent metal coordination, enabling efficient self-assembly^24,25^. While the peptide portion of the peptide-PNA anchors to the QD surface, the PNA binds to complementary nucleic acids via hybridization. PNA is a synthetic analog of DNA with a backbone of N-(2-aminoethyl)-glycine units and nucleobases for base pairing with complementary nucleic acids. As shown in Fig. 1, the PNA binds the dye-labeled hairpin, placing it in proximity to the QD surface to maximize FRET efficiency.

While His-tags have been used extensively in QD-based bioreporters and sensors, including *in cellulo*^26,27^, there have been concerns that the non-covalent nature of His-tag conjugation may limit sensitivity due to dissociation of His-tag bound complexes from QD surfaces upon dilution. Modifying the number and distribution of histidines in His-tags affects the efficiency of protein purification, although limited comparisons are available for QD conjugation^25^. Jiang et al. showed that linear His-tags longer than six histidine residues presented steric hindrance and did not enhance QD-His-tag conjugation^28^. To overcome steric limitations, they designed dendrimeric and cyclic His-tag approaches that successfully improved *K*_D_ but required post-synthesis chemical modifications^29,30^. In pursuit of improved His-tags that did not require additional chemistry beyond solid-state peptide synthesis, we designed and tested four His-tag variations aiming to optimize QD-MB performance (sequences in Supporting Information Table S1, schematics in Fig. 1C). First, the mono-hex His-tag replicates the previous designs with a single six-histidine sequence. Two designs increased the histidine count to 12 with different arrangements: the di-hex His-tag, composed of two six-histidine domains linked by a two-glycine spacer, and the tri-quad His-tag, composed of three four-histidine domains, which was reported to be the smallest domain capable of proper metal coordination^25^. The final His-tag incorporates cysteines preceding and following the six-histidine domain for a dual binding approach, exploiting the His-tag to promote sulfur coordination to the QD surface; this peptide-PNA is called mono + S. The PNA sequence was identical for all designs.

**Fig. 2:**
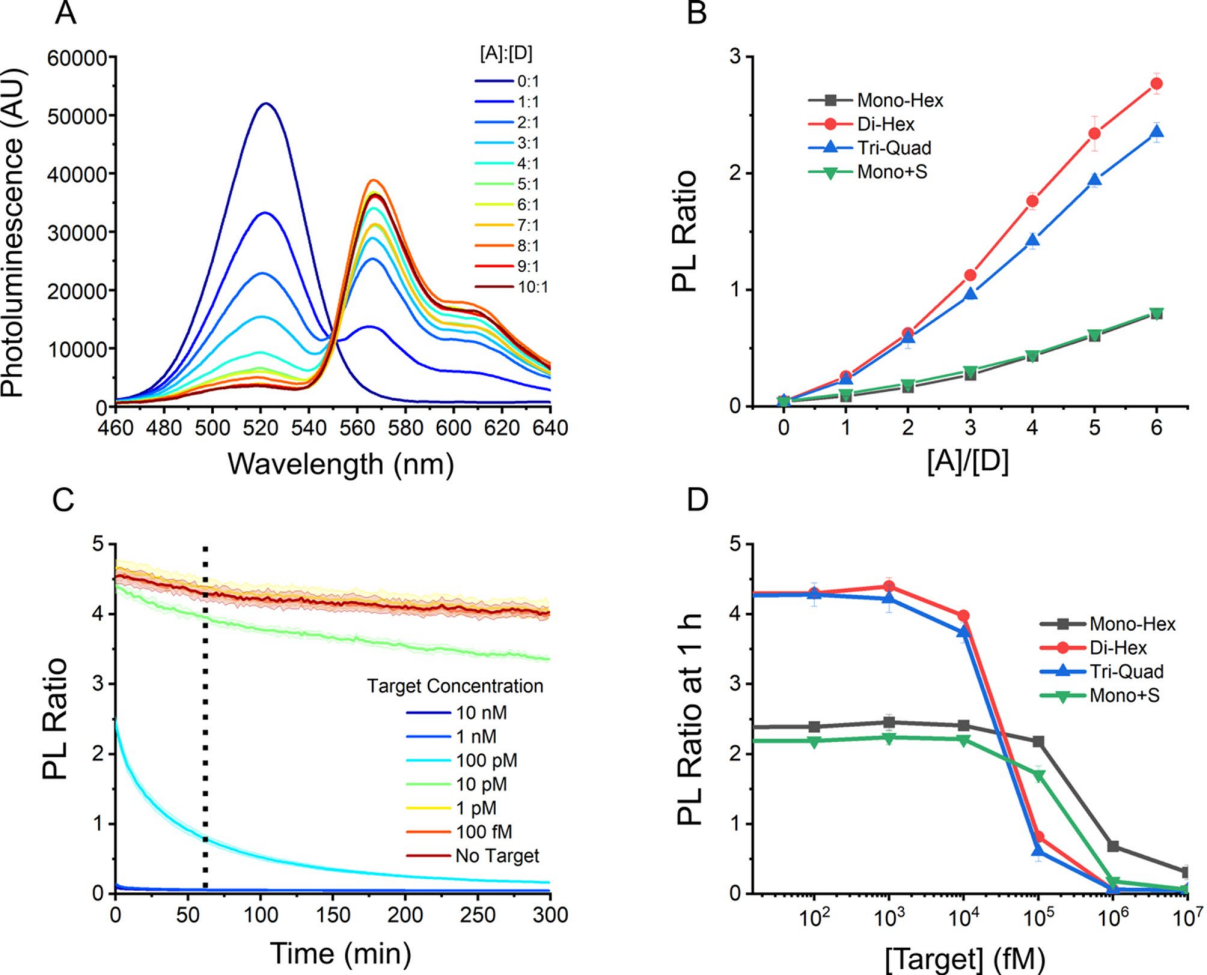
Determining the effects of His-tag design on QD-MB performance. (**A**) Representative fluorescence spectra and (**B**) PL ratios of QD-MBs ([QD] = 200 nM) assembled with varied [A]/[D] molar ratios. QD-MBs were constructed with each of the four His-tag variants: mono-hex, di-hex, tri-quad, and mono + s. Spectra shown in A were collected from QD-MBs assembled with di-hex histags and 0T-8U hairpins. (**C**) CRISPR-Cas RNA detection assay with serially diluted RNA target; time-trace of PL ratio for QD-MBs constructed with di-hex peptide-PNA and 0T-8U hairpins at 6:1 [A]/[D] is shown. (**D**) PL ratios of CRISPR-Cas QD-MB assays using four separate His-tags in a range of target concentrations from 10 nM down to 100 femtomolar (fM) at 60 min.

To compare the efficiency of QD conjugation, each peptide-PNA was assembled with a complementary Cy3-labeled DNA hairpin designed to position Cy3 in proximity to the surface upon conjugation. QDs were then titrated with increasing peptide-PNA/DNA-Cy3 concentrations to achieve varied acceptor to donor ([A]/[D]) molar ratios; representative fluorescence spectra and PL ratios are provided in Fig. 2A and B (raw spectra provided in Fig. S1). FRET efficiency (*E*_FRET_) was plotted against the [A]/[D] ratio, revealing that the three modified His-tags – di-hex, tri-quad, and mono + S – all showed increased *E*_FRET_ over the mono-hex (although the improvement for mono + S was nominal). Assuming an *R*_0_ value of 5.5 nm and using the fitted data provided in Fig. S2, the estimated donor-acceptor distance (*r*_DA_) between the QD and Cy3 ranged from 5.3 ± 0.1 nm for di-hex to 5.7 ± 0.1 nm for mono-hex (center to center). This estimation assumes that all acceptors are bound to donors, and that the number distribution of acceptors per donor is uniform, which is assumed to be approximately valid for the conditions tested. In line with this, the differences in the estimated donor-acceptor distances correlated with the differences in the peptide spacers of each peptide-PNA. To determine the binding affinities of the peptide-PNAs to the QD surface, serial dilutions were performed on the QD-MB complexes ([QD]: [peptide-PNA]: [DHP-Cy3]) assembled at ratios of 1:6:6. QD-MB mixtures were prepared at 200 nM QD and diluted down to as low as 1 nM for FRET measurements, limited by background noise in fluorescence measurements below 1 nM. The results (Fig. S3) confirmed that the di-hex and tri-quad His-tags had significantly higher binding affinities for the QD surface than the mono-hex and mono + S His-tags. The acceptor to donor PL ratio was consistently higher for di-hex and tri-quad, and as the system was diluted, it became more evident, going from a ~ 4-fold difference at 200 nM to a ~ 12-fold difference at 2.5 nM. While we aimed to determine *K*_D_ values from the dilution measurements and FRET calibration curves, we found that the detector sensitivity limits resulted in significant background signals for concentrations below 2.5 nM. All His-tag variations were greater than 50% assembled at 2.5 nM as estimated using PL ratios, in line with the ~ 1 nM *K*_D_ values reported in the literature for hexahistidine tags^25^. The di-hex and tri-quad His-tags still had 66 ± 4% of the ligands bound at 2.5 nM, suggesting that binding affinities in the 10–100 s of pM range are reasonable, although single-molecule or flow-based measurements are required for confirmation.

We then tested whether the differences in peptide-PNA would affect the performance of QD-MBs in Cas13 assays. QD-MBs were assembled with each His-tag variant at a 6:1 [A]/[D] ratio, mixed with preassembled Cas13/gRNA complexes, then titrated with target RNA. Final target RNA concentrations ranged from 10 nM to 100 fM, and the Cas/gRNA complex and QD-MB were held constant at 45 and 100 nM, respectively. Representative PL ratio time-trace curves are shown in Fig. 2C, with D summarizing the results for all peptide-PNA variants after 60 min. An additional summary figure and data are provided in the Supporting Information (Fig. S4), and experimental details are provided in the Materials and Methods. The PL ratio was determined from the ratio of fluorescence intensities measured at 604 and 520 nm, corresponding to the Cy3 acceptor and QD donor fluorescence, respectively. As shown in Fig. 2D, QD-MBs assembled with di-hex and tri-quad His-tags had significantly higher PL ratios when fully assembled than the mono-hex His-tag variants. To quantify the effects of peptide-PNA on limit of detection (LOD), the data shown in Fig. 2D were fitted with a 4-parameter logistic curve, and LOD was determined by the intercept between the fit and the 3.3σ offset from the no-target control (all LOD data is provided in Tables S4 and S5). Crucially, the di-hex and tri-quad His-tags enabled the lowest LODs of 13 ± 5 and 12 ± 2 pM, respectively, while mono-hex and mono + S His-tags had LODs of 98 ± 41 and 79 ± 26 pM, respectively. These results indicate that the di-hex and tri-quad His-tags are optimal, with their improved binding reducing background leakage, likely owing to their larger surface footprint and better QD surface passivation. The di-hex His-tag was selected for further testing because of its qualitatively slightly better *K*_D_ values over the tri-quad. This improved conjugation approach should enable the use of QD-MBs or any QD biosensor assembled via His-tags at lower concentrations, such as those required for in vivo imaging.

### Optimization of substrate hairpin composition

The composition and overall length of reporter substrates have been shown to have a significant effect on the rate of trans-cleavage of Cas13 enzymes^31,32^. Many of these reporters are chimeric in nature, containing both RNA as the active site and DNA as a potential allosteric component. To determine whether the incorporation of these design elements into the hairpin of the QD-MB improves its performance, a pure RNA hairpin and five chimeric DNA-RNA hairpins were designed and characterized using Cas13 assays. The sequences and schematics of the hairpins are available in Supporting Information Table S2 and Fig. 1D. The chimeric hairpins differ only in the loop design; all other design elements are conserved. We note that, in comparison to our previous work^16^, we found that reduction of the stem length from 7 to 5 bp allowed for a more complete release of the cleaved dye-labeled section.

**Fig. 3:**
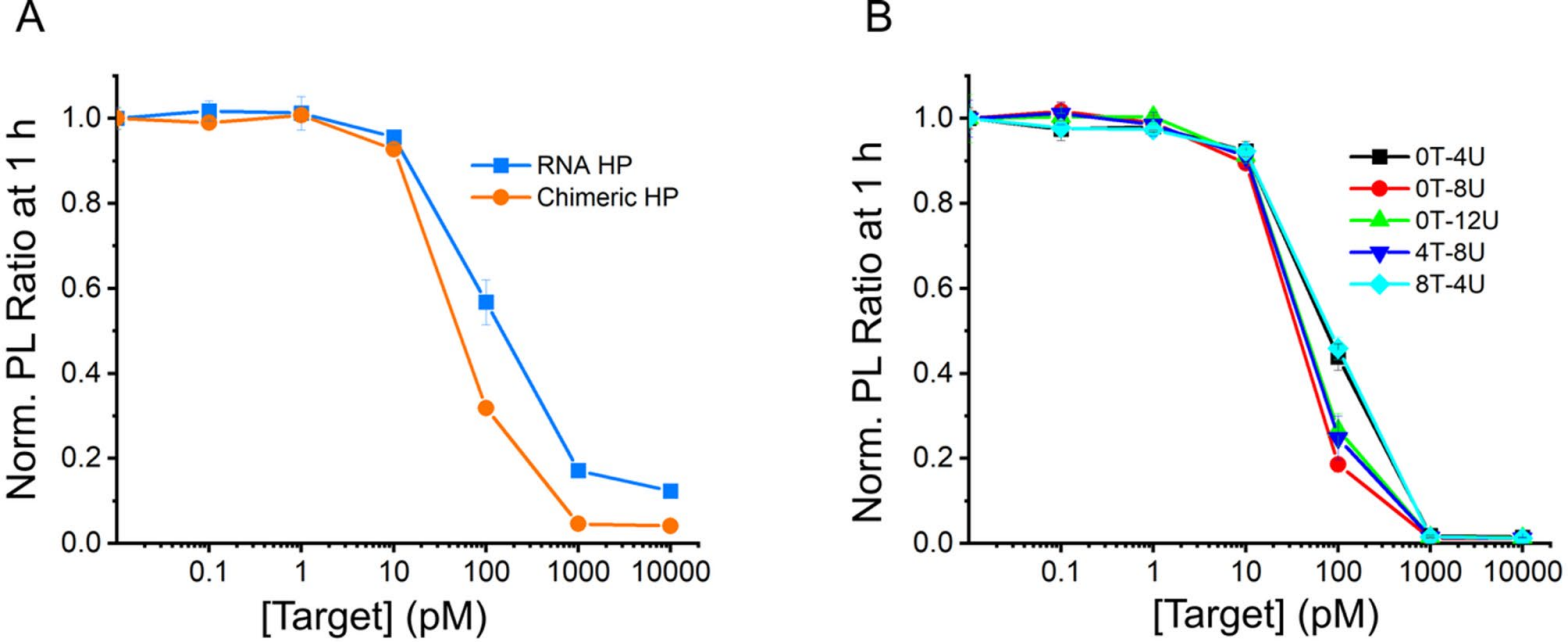
Hairpin composition and length differences in CRISPR-Cas QD-MB assays. (**A**) PL ratios at 60 min of decreasing target concentrations using di-hex His-tags and 0T-12U chimeric hairpins and 12U loop RNA hairpins. (**B**) PL Ratios at 60 min of decreasing target concentrations using di-hex His-tags and five different chimeric hairpins: 0T-4U, 0T-8U, 0T-12U, 4T-8U, 8T-4U.

To optimize signal transduction with QD-MB, we next focused on the nucleic acid hairpin, which is the only component intended to interact directly with Cas enzymes. To determine whether chimeric hairpins improve QD-MB function compared to the traditional full RNA hairpin, we designed and tested a chimeric hairpin with identical length but varied numbers of RNA bases. The results of Cas13 target dilution assays using full RNA and chimeric hairpins are shown in Fig. 3A, with target concentrations ranging from 10 nM to 100 fM. The normalized PL signals clearly demonstrate that the chimeric hairpin provided improved signal transduction with the LOD decreasing from 18 ± 5 pM to 13 ± 5 pM. Comparing the time to half-signal for the 100 pM target, we observed that the chimeric RNA reached this threshold within 8 min, approximately eight times faster than the full RNA hairpin at 67 min. This is significant for real-world applications, particularly point-of-care considerations. Chimeric hairpins showed significant performance improvements and were adopted for further experiments.

Upon selection of chimeric hairpins over RNA analogs, four additional chimeric hairpins were designed with different loop lengths and RNA domain sizes. The chimeric hairpins are depicted in Fig. 1D with poly-uracil (U) RNA domains of varying sizes to satisfy the reported dinucleotide cleavage preferences of LwaCas13a. Hairpin loops containing 4, 8, and 12 U were designed along with two additional hairpins with fixed lengths of 12 nucleotides (nt), but varying numbers of RNA bases. One loop contained 4 T and 8 U while the other contained 8 T and 4 U. Each hairpin was designated according to the constituents of its loop; for example, the loop with four thymine and 8 uracil is identified as “4T-8U.” Thus, the five chimeric hairpins were 0T-4U, 0T-8U, 0T-12U, 4T-8U, and 8T-4U. When present, thymine bases were distributed along the base of the hairpin loop to extend the RNA bases further into the solution and improve steric accessibility. The FRET efficiency and donor-acceptor distances were determined for each of the five hairpins to ensure that the variations did not affect the interpretation of PL ratios measured during Cas assays. As anticipated, there was no statistical difference in the determined *r*_DA_ for the five chimeric hairpins (see Supporting Information, Fig. S5F), all values being either 5.5 ± 0.1 or 5.6 ± 0.1 nm. For target detection with LwCas13a, target concentrations ranging from 10 nM to 100 fM were tested for each hairpin. The results are plotted in Fig. 3B and show that hairpins 0T-8U and 4T-8U possessed slightly lower LODs of 12 ± 16 pM and 15 ± 14 pM, respectively. The remaining three hairpins 0T-4U, 0T-12U, and 8T-4U show LOD of: 20 ± 17, 22 ± 17, and 20 ± 6 pM, respectively. These results suggest that the small 4 nt loop and 4U domain in larger loops may not be ideal for Cas cleavage, in agreement with prior studies^31^, and the best performance was achieved with hairpins containing 8U and larger domains in the loop. Given the lower limit of detection of 0T-8U and the fact that a smaller number of bases in the loop reduces costs and minimizes material that might complicate interactions with the Cas enzyme, it was chosen for subsequent experiments.

### QD surface passivation strategies

**Fig. 4:**
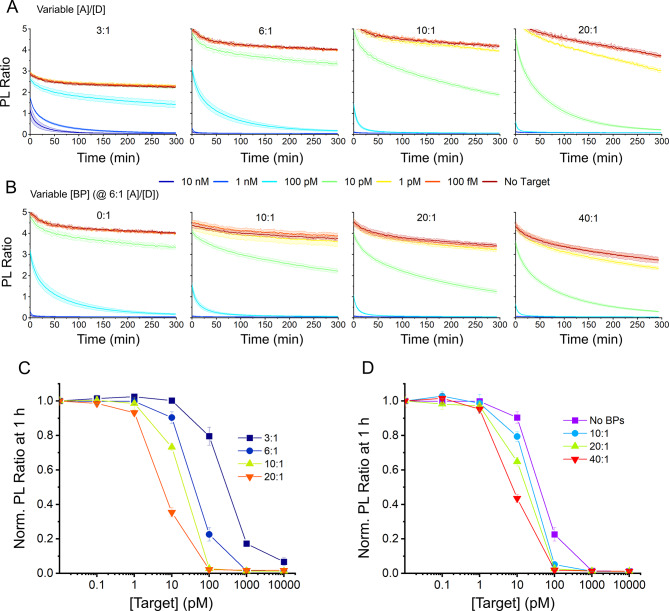
Time traces and LOD data of CRISPR-Cas QD-MB assays while tuning hairpin to quantum dot ([A]/[D]) ratios and blocking peptide (BP) to QD-MB ratios using the 0T-8U dye and di-hex His-tag. (**A**) Time traces of increasing [A]/[D] ratios for the QD-MBs at 3:1, 6:1, 10:1, 20:1. (**B**) Time traces of fixed 6:1 [A]/[D] ratios and varying BP to QD ratios of 0:1, 10:1, 20:1, and 40:1. (**C**) PL ratios plotted against target concentrations for the four [A]/[D] variations shown in (**A**), at 60 min. (**D**) PL ratios plotted against target concentrations for blocking peptide variations shown in (**B**), at 60 min.

After redesigning the His-tag and hairpin loop, the focus was shifted to the quantity of the hairpin used. It was speculated that some of the Cas enzyme population became bound to QDs upon addition of QD-MBs, resulting in enzymatic inactivation as demonstrated previously^33^. While the results in Fig. 1 suggested that the QD surface approached saturation at 6:1 di-hex and tri-quad His-tags, the non-covalent nature of the His-tag-QD bond might result in exchange of peptide-PNA/hairpin complexes with Cas enzymes over time. To determine if this exchange was indeed occurring and limiting the sensitivity of our assays, four different [A]/[D] ratios of 3:1, 6:1, 10:1, and 20:1 were tested. At [A]/[D] ratios above the suspected His-tag saturation point^34^, i.e., 10:1 and 20:1, hairpin/peptide-PNA complexes would be available to compete with Cas enzymes to bind any available sites on the QD surface, reducing the amount of Cas lost to adsorption onto QDs and thus improving Cas activity and assay sensitivity. The results, shown in Fig. 4, supported this hypothesis; Cas activity and assay sensitivity increased significantly with greater [A]/[D] ratios despite the increased background noise anticipated for higher acceptor concentrations. The LODs for 3:1, 6:1, 10:1, and 20:1 [A]/[D] ratios at 60 min were determined to be 46 ± 48, 9 ± 9, 4 ± 1, and 1.8 ± 0.3 pM, respectively.

To further confirm that the sensitivity increase at higher [A]/[D] ratios was due to QD surface passivation, excess peptide-PNA/hairpin complexes were exchanged for blocking peptides (BPs), i.e., short peptide strands (NH_2_-H_6_GWD_3_-COOH) that were previously developed to passivate the ZnS surface of QDs against protein binding^33^. Four BP-to-QD ratios were tested: 0:1, 10:1, 20:1, and 40:1, with [A]/[D] ratio constant at 6:1. QD-MB concentrations were held constant at 100 nM as in prior experiments, and the BPs were thus introduced at 0, 1000, 2000, and 4000 nM. The resulting PL ratio time traces are shown in Fig. 4B, with D summarizing the effects of BPs on the assay LOD. The LODs were determined to be 9 ± 9, 4.4 ± 0.5, 4 ± 2, and 3.1 ± 0.1 pM for BP: QD ratios of 0:1, 10:1, 20:1, and 40:1, respectively. The improvement in sensitivity with greater BP: QD ratios further confirmed our hypothesis that Cas activity was hindered by unintentional binding of Cas enzyme to the QD-MBs at low [A]/[D] ratios.

### Testing additional excipients and optimization

We aimed to take the lessons learned from the experiments described above and determine what was the minimum detectable target concentration for LwCas13a with QD-MB readout. To supplement these experiments, we tested previously reported approaches to improve Cas detection limits with excipients, including: Tris buffer concentration^35^, synergistic salt addition^36^, and the inclusion of surfactants or poly-T DNA strands^37^. For QD-MB assays, none of these additives resulted in detectable improvements (See Figs. S6, S7) and as such were omitted to simplify assay conditions. The use of freshly ligand-exchanged QDs provided some notable improvement in performance (Fig. S8), though for the sake of consistency, the original QD mixture was used for all experiments shown in the main text. An attempt at an optimized assay using di-hex His-Tags, 0T-8U hairpins, and 40:1 [A]/[D], shown in Fig. S9, achieved target detection down to 250 fM, though it underperformed other experiments at 60 min and required 5 h run time to achieve the 250 fM detection. This result was nonetheless promising, particularly because Cas activity was maintained for the entire run and continued to demonstrate substrate cleavage even at 100 fM target. This suggests that the current LODs are limited by the kinetics of LwCas13a trans-cleavage rather than insufficient sensitivity of enzyme-target binding. Further, the continued activity of the Cas enzyme and functionality of the QD-MBs after 5 h suggests that the previously observed enzyme deactivation was not due to a natural upper limit on the number of turnover events. Rather, prolonged Cas activity in the presence of fully passivated QDs (40:1 [A]: [D]) suggests that the previously observed Cas inactivation was QD-surface induced (see Green et al. 2023)^33^.

### Portable Lamp-and-Phone detection

**Fig. 5:**
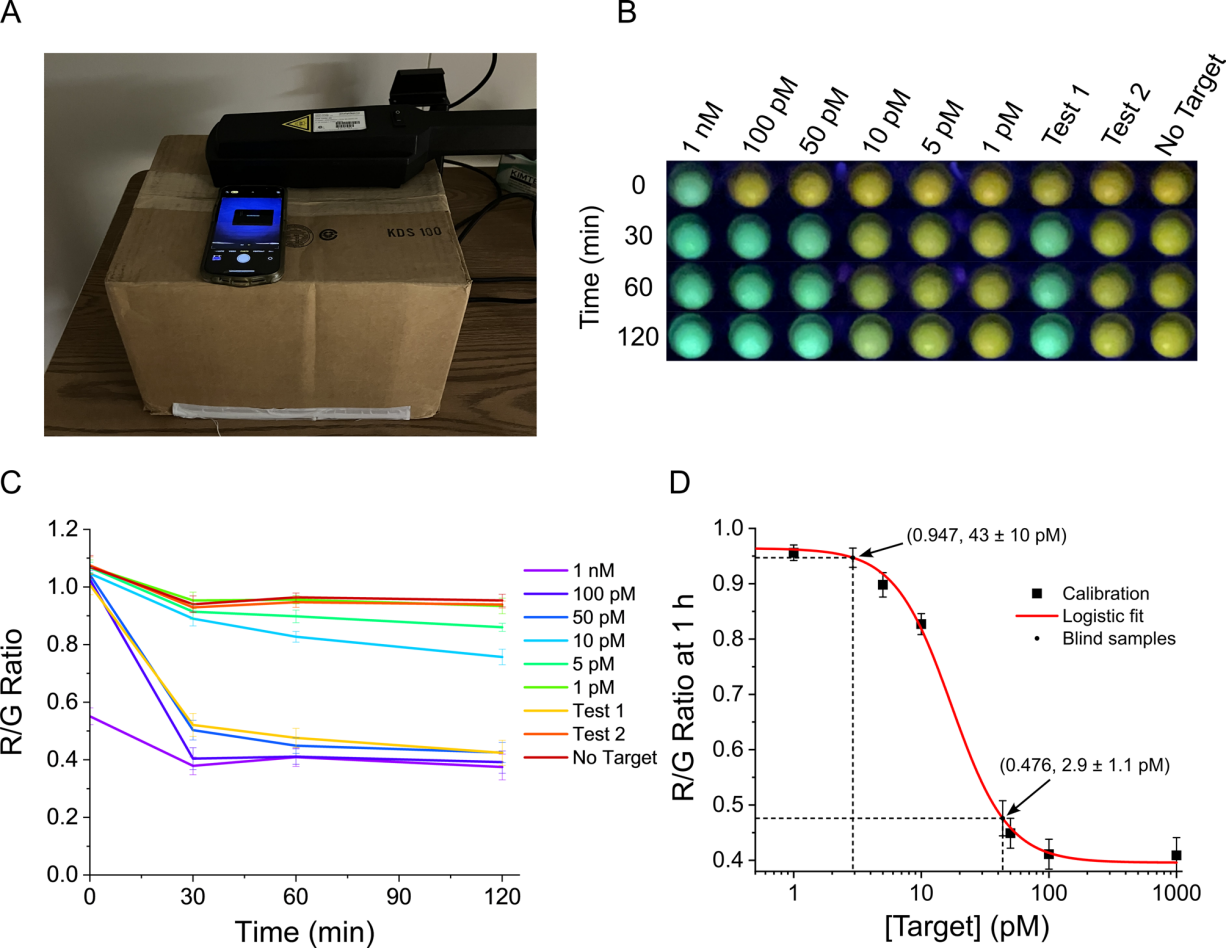
Countertop experiment completed with UV-lamp and cell phone camera. (**A**) An image of the countertop experiment depicts a box covering a well-plate, a UV-lamp exciting the QD-MBs into fluorescence, and a camera-phone to capture the images through an opening at the top of the box. (**B**) Seven known and two mystery target concentrations were tested with CRISPR-Cas QD-MB assays to demonstrate field-forward quantitative detection and images were taken of side-by-side samples at 0, 30, 60 and 120 min. (**C**) Red image intensities were divided by green image intensities from (**B**) to obtain PL ratios for seven known target concentrations and two mystery concentrations. (**D**) The data of the seven known targets concentrations taken from (**C**) at 60 min were plotted according to their PL Ratios and a logistic fit used to determine mystery concentrations via dashed lines from their measured fluorescence.

An important aspect in making CRISPR/Cas assays field portable is minimizing the requirements for complex lab equipment for determination. While we previously reported 5 nM target detection using a UV lamp and the camera from a cellphone, we wished to determine if the low-tech assay could be improved to offer picomolar LOD while maintaining the quantitative nature. For this experiment, QD-MBs with di-hex histags and 0T-8U hairpins were used at [A]/[D] of 20:1. A calibration set containing seven known concentrations were run next to two unknown test samples to determine if a second, uninformed user could predict sample concentrations using a calibration curve generated from the standards. Target concentrations for the calibration curve ranged from 1 nM to 1 pM (with negative control) and are indicated in Fig. 5B. Once the reactions were initiated, the plate was imaged with a cellphone camera under a UV lamp in a dark room (Fig. 5A), then covered and incubated at 25 °C. The microplate was imaged again at 30, 60, and 120 min; raw images are provided in Fig. S10. To determine PL ratios from the images, red and green channels were extracted from the RGB images, and the PL ratio was calculated by the ratio of red/green pixel intensities. The results for each time point are shown in Fig. 5C, and the fitted calibration curve at 60 min is shown in Fig. 5D. Using the calibration curve, the blind samples were estimated to be 2.9 ± 1.1 pM and 43 ± 10 pM by the second user, within about 10% of the actual concentrations of 2.5 and 40 pM, respectively. Test sample 2 had a target concentration below the calculated LOD of 4.3 ± 1.2 pM for the assay, thus it was not a statistically significant detection despite the close estimate. These results demonstrate the utility of CRISPR-Cas QD-MBs in field-forward compatible formats and motivate incorporation into deployable assays.

## Conclusion

Rapid, amplification-free CRISPR diagnostics that can be employed outside the laboratory remains a long-standing goal. By re-engineering the individual components of the quantum dot molecular beacon (QD-MB) – (i) multivalent His-tags with greater ZnS affinity to drive donor-acceptor assembly and (ii) chimeric DNA-RNA hairpins that accelerate LwCas13a trans-cleavage and reduce sensitivity to RNase contamination – and further optimizing the conditions for QD-MB assembly and CRISPR assays, we cut the limit-of-detection (LOD) from roughly 100 pM down to ~ 1 pM without target amplification. Under smartphone imaging and inexpensive UV illumination, we demonstrated quantitative readout to below 10 pM, a significant step towards fieldable diagnostics that was achieved through optimization of signal transduction alone.

While the performance demonstrated here sits well above many clinically relevant viral RNA titers, we anticipate that the combination of QD-MBs with engineered Cas13 enzymes and multivalent gRNA pools would reduce LOD significantly while maintaining the advantageous aspects of the assays demonstrated here. Recent work by Yang et al.^35^ demonstrated ~ 10 fM target detection using an engineered LwCas13a containing addition RNA binding domains, and prior work by Nalefski et al.^31^ demonstrated a 10-fold improvement in Cas13a activity with the use of an optimized pool of guide RNA designed to tile a single target. Further development of reagent lyophilization strategies and shelf-stable preparations are needed to propel the QD-MB out of the lab toward commercialization. However, the work here lays a foundation for lightweight, low complexity quantitative diagnostics capable of tracking pathogens in real time.

## Materials & methods

Quantum-dot molecular beacons were assembled using three separate components: quantum dots, peptide/PNA chimeric His-tags, and RNA hairpins or RNA/DNA chimeric hairpins. Quantum dots were synthesized in-house with a CdSe core and CdS/ZnS shell. Ligand exchange was performed prior to use of QDs for solubility in aqueous conditions. More information on quantum dot synthesis can be found in the supporting information of Green et al.^16^. The peptide-PNA chimeric His-tags (Table S1) were ordered from PNA Bio. RNA hairpins and DNA/RNA chimeric hairpins (Table S2) were ordered from Integrated DNA Technologies (IDT). Before use, the QD stock solution (post ligand exchange, stored at 4 °C) was sonicated for 5–10 min to break up any small aggregates formed during storage. The QDs were diluted to a working concentration of 2 µM. Hairpins were combined with peptide/PNA His-tags at a 1:1 ratio at 20 µM in 50 mM Tris at pH of 8.0), 50 mM NaCl, and 10 mM MgCl_2_ buffer before annealing in a PCR tube (70–20 °C, cooled at 2.5 °C/min) to drive hybridization between the 14 nt PNA domain and the complementary domain on the hairpin strands. These working solutions were combined in diluted mixtures to assemble QD-MBs for Cas assays.

LwCas13a with C-terminal six-histidine tags were expressed in-house in BL21 Star (DE3) *E. coli* from Invitrogen using pET30a-LwCas13a plasmid obtained from GenScript, then purified and concentrated with Ni-NTA IMAC (immobilized metal affinity chromatography) columns and dialysis to remove excess imidazole. The final protein stocks were prepared in aliquots containing 18 µM protein in 50 mM Tris-HCl pH 7.5, 600 mM NaCl, and 5% glycerol, and flash frozen prior to storage at −80 °C. LwCas13a aliquots were thawed immediately prior to use, and fresh 250 mM TCEP was added to thawed aliquots for a final concentration of 10 mM TCEP. The aliquots were then incubated at 37 °C for 15 min to deactivate any RNases carried over from expression. This mixture was then used to assemble Cas and guide RNA complexes.

For target dilution assays, target strands were serially diluted in water by factors of 10 or less, from 40 nM to 400 fM. The Cas13 protein, guide RNA, and QD-MB solutions were pipetted into the wells of a Corning 384 round well, low volume, and clear bottom plate. Target solutions were then pipetted into wells starting with the lowest concentrations and finishing with the highest concentration to capture the kinetics of the faster reactions. The final solutions contained 30 µL of 100 nM QD, varied concentrations of hairpin complex (but often 600 nM for 6:1 [A]/[D]), 50 nM Cas13, 45 nM guide RNA, 50 mM Tris at pH 8.0, 50 mM NaCl, 10 MgCl_2_, and varying amounts of target RNA, diluted 4-fold from the as-prepared serial dilutions. Target RNA was represented by a short 35 nt domain from *lcrV*, and the guide RNA included a 28 nt spacer that was fully complementary to 28 nt of the target (Table S3). Plates were covered with Applied Biosystems MicroAmp™ optical adhesive film to reduce evaporation during data collection. Data were collected via TECAN Spark fluorescent plate readers exciting the QDs at 380 nm wavelength and collecting either emission at various wavelengths or emission wavelengths of 520 nm and 604 nm for kinetics experiments. An emission wavelength of 604 nm was found to be the optimal collection wavelength, according to our previous work.

For experiments using blocking peptides (Table S1), blocking peptide was added 45 min after QD-MB assembly and allowed to assemble at room temperature for 1 h prior to use. Blocking peptide stocks were stored at 500 µM in 10% DMSO at –20 °C, and blocking peptide was added directly to QD-MB stock without pre-dilution to minimize dilution of the prepared QD-MB complexes. Excipients intended to reduce non-specific binding, such as surfactants and poly-T DNA, were added to target mixtures prior to serial dilution and additionally included in the solutions used for dilutions, at the intended final concentrations. For divalent salt excipients, these were added to the Cas/gRNA complex mixture and included in the final reaction mixture at the final intended concentrations. All excipient concentrations were carefully controlled to avoid exposing any enzyme or nucleic acid to concentrated mixtures of the excipients.

## Electronic supplementary material

Below is the link to the electronic supplementary material.

Supplementary Material 1

## Data Availability

All data used in this study and not included in the supplementary materials are available from the corresponding authors upon request.

## References

[1] Jansen, R., Embden, J. D., Gaastra, W. & Schouls, L. M. Identification of genes that are associated with DNA repeats in prokaryotes. *Mol. Microbiol.* **43**, 1565–1575. 10.1046/j.1365-2958.2002.02839.x (2002). 11952905 10.1046/j.1365-2958.2002.02839.x

[2] Barrangou, R. et al. CRISPR provides acquired resistance against viruses in prokaryotes. *Science* **315**, 1709–1712. 10.1126/science.1138140 (2007). 17379808 10.1126/science.1138140

[3] Mojica, F. J., Díez-Villaseñor, C., García-Martínez, J. & Soria, E. Intervening sequences of regularly spaced prokaryotic repeats derive from foreign genetic elements. *J. Mol. Evol.* **60**, 174–182. 10.1007/s00239-004-0046-3 (2005). 15791728 10.1007/s00239-004-0046-3

[4] Bolotin, A., Quinquis, B., Sorokin, A. & Ehrlich, S. D. Clustered regularly interspaced short palindrome repeats (CRISPRs) have spacers of extrachromosomal origin. *Microbiology* **151**, 2551–2561. 10.1099/mic.0.28048-0 (2005). 16079334 10.1099/mic.0.28048-0

[5] Deveau, H., Garneau, J. E. & Moineau, S. CRISPR/Cas system and its role in phage-bacteria interactions. *Annu. Rev. Microbiol.* **64**, 475–493. 10.1146/annurev.micro.112408.134123 (2010). 20528693 10.1146/annurev.micro.112408.134123

[6] Brouns, S. J. et al. Small CRISPR RNAs guide antiviral defense in prokaryotes. *Science* **321**, 960–964. 10.1126/science.1159689 (2008). 18703739 10.1126/science.1159689 PMC5898235

[7] Makarova, K. S. et al. Evolution and classification of the CRISPR-Cas systems. *Nat. Rev. Microbiol.* **9**, 467–477. 10.1038/nrmicro2577 (2011). 21552286 10.1038/nrmicro2577 PMC3380444

[8] Makarova, K. S. et al. An updated evolutionary classification of CRISPR–Cas systems. *Nat. Rev. Microbiol.* **13**, 722–736. 10.1038/nrmicro3569 (2015). 26411297 10.1038/nrmicro3569 PMC5426118

[9] Abudayyeh, O. O. et al. C2c2 is a single-component programmable RNA-guided RNA-targeting CRISPR effector. *Science* **353**, aaf5573. 10.1126/science.aaf5573 (2016). 27256883 10.1126/science.aaf5573 PMC5127784

[10] Koonin, E. V., Makarova, K. S. & Zhang, F. Diversity, classification and evolution of CRISPR-Cas systems. *Curr. Opin. Microbiol.* **37**, 67–78. 10.1016/j.mib.2017.05.008 (2017). 28605718 10.1016/j.mib.2017.05.008 PMC5776717

[11] Chen, J. S. et al. CRISPR-Cas12a target binding unleashes indiscriminate single-stranded DNase activity. *Science* **360**, 436–439. 10.1126/science.aar6245 (2018). 29449511 10.1126/science.aar6245 PMC6628903

[12] Tang, Y. et al. The CRISPR–Cas toolbox for analytical and diagnostic assay development. *Chem. Soc. Rev.* **50**, 11844–11869. 10.1039/D1CS00098E (2021). 34611682 10.1039/d1cs00098e

[13] Gootenberg, J. S. et al. Nucleic acid detection with CRISPR-Cas13a/C2c2. *Science* **356**, 438–442. 10.1126/science.aam9321 (2017). 28408723 10.1126/science.aam9321 PMC5526198

[14] East-Seletsky, A. et al. Two distinct RNase activities of CRISPR-C2c2 enable guide-RNA processing and RNA detection. *Nature* **538**, 270–273. 10.1038/nature19802 (2016). 27669025 10.1038/nature19802 PMC5576363

[15] Huang, D. et al. Advances and challenges of signal readout systems in CRISPR-based biosensors for point-of-care testing of nucleic acid. *TrAC Trends Anal. Chem.* **178**, 117856 (2024). 10.1016/j.trac.2024.117856

[16] Green, C. M. et al. Quantum dot-based molecular beacons for quantitative detection of nucleic acids with CRISPR/Cas(N) nucleases. *ACS Nano*. **16**, 20693–20704. 10.1021/acsnano.2c07749 (2022). 36378103 10.1021/acsnano.2c07749

[17] Medintz, I. L., Uyeda, H. T., Goldman, E. R. & Mattoussi, H. Quantum dot bioconjugates for imaging, labelling and sensing. *Nat. Mater.* **4**, 435–446. 10.1038/nmat1390 (2005). 15928695 10.1038/nmat1390

[18] Medintz, I. L. et al. Self-assembled nanoscale biosensors based on quantum dot FRET donors. *Nat. Mater.* **2**, 630–638. 10.1038/nmat961 (2003). 12942071 10.1038/nmat961

[19] Dos Santos, M. C., Algar, W. R. & Medintz, I. L. Hildebrandt, N. Quantum dots for Förster resonance energy transfer (FRET). *TrAC Trends Anal. Chem.* **125**, 115819. 10.1016/j.trac.2020.115819 (2020).

[20] Hildebrandt, N. et al. Energy transfer with semiconductor quantum Dot bioconjugates: A versatile platform for biosensing, energy harvesting, and other developing applications. *Chem. Rev.* **117**, 536–711 (2017). 27359326 10.1021/acs.chemrev.6b00030

[21] Green, C. M. et al. Factors limiting the sensitivity of DNA-conjugated quantum dot molecular beacons. In *Colloidal Nanoparticles for Biomedical Applications XVIII* (eds Osiński, M. & Kanaras, A. G.) *Proc. SPIE* **12395**, 1239505. 10.1117/12.2649233 (2023).

[22] Susumu, K. et al. Multifunctional compact zwitterionic ligands for preparing robust biocompatible semiconductor quantum dots and gold nanoparticles. *J. Am. Chem. Soc.* **133**, 9480–9496. 10.1021/ja201919s (2011). 21612225 10.1021/ja201919s

[23] Green, C. M. et al. Polyhistidine-tag-enabled conjugation of quantum dots and enzymes to DNA nanostructures, in *Bioluminescence: Methods and Protocols* Vol. 2 61–91 (eds Kim, S. B.) (Springer, Cham, 2022). 10.1007/978-1-0716-2473-9_6 35836061

[24] Blanco-Canosa, J. B. et al. Recent progress in the bioconjugation of quantum dots. *Coord. Chem. Rev.* **263–264**, 101–137. 10.1016/j.ccr.2013.08.030 (2014).

[25] Sapsford, K. E. et al. Kinetics of metal-affinity driven self-assembly between proteins or peptides and CdSe – ZnS quantum dots. *J. Phys. Chem. C*. **111**, 11528–11538. 10.1021/jp073550t (2007).

[26] Tosat-Bitrián, C. et al. Membrane-targeted quantum dot-based BACE1 activity sensors for in vitro and in cellulo assays. *ACS Appl. Mater. Interfaces*. **16**, 63186–63194. 10.1021/acsami.4c12560 (2024). 39515812 10.1021/acsami.4c12560 PMC11583121

[27] Breger, J. C. et al. Quantum dot lipase biosensor utilizing a custom-aynthesized peptidyl-ester substrate. *ACS Sens.* **5**, 1295–1304. 10.1021/acssensors.9b02291 (2020). 32096987 10.1021/acssensors.9b02291

[28] Wang, J. et al. Capillary electrophoretic studies on quantum dots and histidine appended peptides self-assembly. *Electrophoresis* **36**, 2419–2424. 10.1002/elps.201500205 (2015). 26084876 10.1002/elps.201500205

[29] Qiu, L. et al. De Novo design of a cyclic polyhistidine peptide for binding with quantum dots: self-assembly investigation using capillary electrophoresis. *Chromatographia* **81**, 41–46. 10.1007/s10337-017-3319-x (2017).

[30] Wang, J. & Xia, J. Preferential binding of a novel polyhistidine peptide dendrimer ligand on quantum dots probed by capillary electrophoresis. *Anal. Chem.* **83**, 6323–6329. 10.1021/ac2011922 (2011). 21728332 10.1021/ac2011922

[31] Nalefski, E. A. et al. Kinetic analysis of Cas12a and Cas13a RNA-guided nucleases for development of improved CRISPR-based diagnostics. *iScience* **24**, 102996. 10.1016/j.isci.2021.102996 (2021). 34505008 10.1016/j.isci.2021.102996 PMC8411246

[32] Zhang, D. et al. CRISPR/Cas12a-mediated interfacial cleaving of hairpin DNA reporter for electrochemical nucleic acid sensing. *ACS Sens.* **5**, 557–562. 10.1021/acssensors.9b02461 (2020). 32013399 10.1021/acssensors.9b02461

[33] Green, C. M. et al. Passivating quantum dots against histag-displaying enzymes using blocking peptides: salient considerations for self-assembling quantum dot biosensors. *Sens. Diagn.* **2**, 1521–1530. 10.1039/d3sd00149k (2023).

[34] Prasuhn, D. E. et al. Polyvalent display and packing of peptides and proteins on semiconductor quantum dots: Predicted versus experimental results. *Small* **6**, 555–564. 10.1002/smll.200901845 (2010). 20077423 10.1002/smll.200901845

[35] Yang, J. et al. Engineered LwaCas13a with enhanced collateral activity for nucleic acid detection. *Nat. Chem. Biol.* **19**, 45. 10.1038/s41589-022-01135-y (2023). 36138140 10.1038/s41589-022-01135-y

[36] Feng, Z. Y., Yun, Y. F., Li, X. & Zhang, J. J. Impact of divalent metal ions on regulation of trans-cleavage activity of CRISPR-Cas13a: A combined experimental and computational study. *ChemBioChem* **24**, e202300034 (2023). 10.1002/cbic.202300034 37040174 10.1002/cbic.202300034

[37] Zhang, D. Y., Turberfield, A. J., Yurke, B. & Winfree, E. Engineering entropy-driven reactions and networks catalyzed by DNA. *Science* **318**, 1121–1125. 10.1126/science.1148532 (2007). 18006742 10.1126/science.1148532

