# Supplementary material for: Quantum dot molecular beacons achieve sub-10 pM CRISPR-Cas detection in field-ready assays
Source: Sci Rep. 2025 Jul 31;15:27950. doi: 10.1038/s41598-025-09434-9 (PMC12313975; doi:10.1038/s41598-025-09434-9)
Supplement: Supplementary file 1 — Supplementary Material 1 [file 41598_2025_9434_MOESM1_ESM.pdf]

Supporting Information

Quantum dot molecular beacons achieve sub-10 pM CRISPR-Cas detection in field-ready assays

Drew P. Lysne[1,2], Michael H. Stewart[3], Kimihiro Susumu[3], Tomasz A. Leski[2], David A. Stenger[2], Igor L. Medintz[2], Sebastián A. Díaz[2], Christopher M. Green[2*]*

[1] National Research Council, 500 Fifth St NW, Washington, DC 20001, United States
[2] Center for Bio/Molecular Science and Engineering, U.S. Naval Research Laboratory Code 6900, Washington, DC 20375, United States
[3] Optical Sciences Division, Code 5600, U.S. Naval Research Laboratory, Washington, DC 20375, United States

*sebastian.a.diaz2.civ@us.navy.mil; christopher.m.green127.civ@us.navy.mil

Table S1 - Peptide/PNA sequences of the four his-tags variants tested in this work. The transition from amino acids (left) to PNA bases (right) is indicated by a colon. The blocking peptide sequence is also provided.

Strand Name	Sequence (N-C, 5'-3')
Mono-Hex His-tag	HHHHHHEDDRED:TCTACTATCTCATC
Di-Hex His-tag	HHHHHHGGGHHHHHHGADD:TCTACTATCTCATC
Tri-Quad His-tag	HHHHGGHHHHGGHHHHGADD:TCTACTATCTCATC
Mono+S His-tag	CGHHHHHHGCAAD:TCTACTATCTCATC
Blocking peptide	HHHHHHGWDDD

Table S2 - Chimeric hairpin and RNA hairpin sequences with DNA bases denoted as A, T, C, G and RNA bases as rA, rU, rC, and rG. A 3' terminal Cy3 dye is denoted by /3CySp/.

Strand Name	Sequence (5’ – 3’)
0T-4U	GATGAGATAGTAGACCCTCTCrUrUrUrUGAGAG/3Cy3Sp/
0T-8U	GATGAGATAGTAGACCCTCTCrUrUrUrUrUrUrUGAGAG/3Cy3Sp/
0T-12U	GATGAGATAGTAGACCCTCTCrUrUrUrUrUrUrUrUrUrUrUGAGAG/3Cy3Sp/
4T-8U	GATGAGATAGTAGACCCTCTCTTrUrUrUrUrUrUrUTTGAGAG/3Cy3Sp/
8T-4U	GATGAGATAGTAGACCCTCTCTTTTrUrUrUrUTTTTGAGAG/3Cy3Sp/
RNA Hairpin	rGrArUrGrArGrArUrArGrUrArGrArCrCrCrUrCrUrCrUrUrUrUrUrUrUrUrUrUrUrUrGrArGrArG/3Cy3Sp/

Table S3 - Target and guide RNA sequences for Cas13 protein used in all assays.

Strand Name	Sequence (5’ – 3’)
RNA Target Strand IcrV-35nt	UUCAGUUAGUCAAAGAUAAAAAUAUAGAUAUUUCC
gRNA Strand	GGGGAUUUAGACUACCCCAAAAACGAAGGGGACUAAAACAAUAUCUAUAUUUUUAUCUUUGACUAAC

Table S4 – Experimental conditions and corresponding limit-of-detection

**Sample**	**Figure**	**HP type**	**HP comp**	**Histag**	**[HP]/[QD]**	**[BP]/[QD]**	**LOD (pM)**	**95% CI Upper**	**95% CI Lower**
Original RHP-QD*	3F [1]	RNA	15U (7 nt stem)	Mono-Hex	6	40	82 ± 73	37 pM	183 pM
RNA 12U	3A	**RNA**	**12U**	Di-Hex	6	0	18 ± 5	14 pM	23 pM
Mono-Hex	2D	Chimeric	0T-8U	**Mono-Hex**	6	0	98 ± 41	69 pM	151 pM
Di-Hex	2D	Chimeric	0T-8U	**Di-Hex**	6	0	13 ± 5	10 pM	19 pM
Tri-Quad	2D	Chimeric	0T-8U	**Tri-Quad**	6	0	12 ± 2	10 pM	13 pM
Mono+S	2D	Chimeric	0T-8U	**Mono+S**	6	0	79 ± 26	59 pM	111 pM
0T-4U	3B	Chimeric	**0T-4U**	Di-Hex	6	0	20 ± 17	14 pM	47 pM
0T-8U	3A,B	Chimeric	**0T-8U**	Di-Hex	6	0	12 ± 16	8 pM	39 pM
0T-12U	3B	Chimeric	**0T-12U**	Di-Hex	6	0	22 ± 17	14 pM	48 pM
4T-8U	3B	Chimeric	**4T-8U**	Di-Hex	6	0	15 ± 14	10 pM	38 pM
8T-4U	3B	Chimeric	**8T-4U**	Di-Hex	6	0	20 ± 6	16 pM	27 pM
3:1 Di-Hex/0T-8U	4C	Chimeric	0T-8U	Di-Hex	**3**	0	46 ± 48	23 pM	118 pM
6:1 Di-Hex/0T-8U	4C	Chimeric	0T-8U	Di-Hex	**6**	0	9 ± 9	6 pM	24 pM
10:1 Di-Hex/0T-8U	4C	Chimeric	0T-8U	Di-Hex	**10**	0	4 ± 1	4.0 pM	6.0 pM
20:1 Di-Hex/0T-8U	4C	Chimeric	0T-8U	Di-Hex	**20**	0	1.8 ± 0.3	1.8 pM	3.0 pM
10:1 BP:QD	4D	Chimeric	0T-8U	Di-Hex	6	**10**	4.4 ± 0.5	4.0 pM	5.0 pM
20:1 BP:QD	4D	Chimeric	0T-8U	Di-Hex	6	**20**	4 ± 2	3.0 pM	6.0 pM
40:1 BP:QD	4D	Chimeric	0T-8U	Di-Hex	6	**40**	3.1 ± 0.1	3.0 pM	3.1 pM
**Tabletop Assay**	5D	Chimeric	0T-8U	Di-Hex	20	**0**	4 ± 1	3.3 pM	5.6 pM
6:1 Di-Hex/0T-8U**	--	Chimeric	0T-8U	Di-Hex	6	0	4 ± 1	3.0 pM	5.0 pM
40:1 BP:QD**	--	Chimeric	0T-8U	Di-Hex	6	40	1.0 ± 0.2	0.8 pM	1.2 pM
40:1 Mono-Hex**	--	Chimeric	0T-8U	Mono-Hex	40	0	4.6 ± 0.9	3.7 pM	5.5 pM
20:1 Tri-Quad**	--	Chimeric	0T-12U	Tri-Quad	20	0	0.9 ± 0.1	0.8 pM	1.0 pM
6:1 0T-12U**	--	Chimeric	0T-12U	Di-Hex	6	0	3.2 ± 0.5	3.0 pM	4.0 pM

*Green *et al.*, 2022 [1] **performed with new batch of QDs

Experiments were run in triplicate, and experimental uncertainty was determined from variations in PL ratio between replicates, and PL ratios were normalized to the control for further analyses. Limit-of-detection (LOD) values for all experimental conditions were calculated after 60 min by fitting the data to a four-parameter logistic (4PL) model; the LOD is the analyte concentration (x_{LOD}) whose fitted signal equals the mean response of the negative-control baseline (y_c) minus 3.3 σ_c (σ_c = standard deviation of the negative-control response), i.e., $y_{LOD} \equiv y_c - 3.3\sigma_c$. The 4PL model is provided in Eq. 1 below, where A_1 (upper asymptote), A_2 (lower asymptote), x_0 (inflection point), and p (shape factor) represent the four parameters to be optimized. Given that all samples were normalized such that the negative control signal (y_c) equals 1 and should only decrease if target is present, the upper asymptote was fixed ($A_1 = 1$) for all samples, reducing the number of fitted parameters to 3.

$$y_{LOD} = \frac{(A_1 - A_2)}{1 + \left(\frac{x_{LOD}}{x_0}\right)^p} \tag{1}$$

To solve for the analyte concentration LOD (x_{LOD}), y_{LOD} is substituted and the equation is rearranged (Eq. 2 below).

$$x_{LOD} = x_0 \left(\frac{(A_1 - A_2)}{(y_c - 3.3\sigma_c - A_2)} - 1 \right)^{\frac{1}{p}} \quad (2)$$

4PL models were fitted to the normalized PL ratios vs target concentration (at 60 min) for every sample using OriginPro data analysis and graphing software. The 95% confidence bands were derived from the fitted parameters and used to determine the upper and lower bounds of the 95% confidence interval for the LOD using OriginPro's built-in utility to derive X from Y, and those values are provided in Table S4. Error reported for the LOD ($x_{LOD} \pm \sigma_{LOD}$) is approximate since the uncertainty in 4PL models is asymmetric around the mean, and σ_{LOD} is only intended to provide a quick approximation of the uncertainty for readers in the main text. The fitted 4PL parameters for every sample are provided below.

Table S5 – Samples and corresponding fitted parameters of 4PL model

Sample	A_1	SE	A_2	SE	x_0	SE	p	SE	σ_c
Original RHP-QD*	1.000	--	0.538	0.024	902	141	1.04	0.2	0.011
RNA 12U	1.000	--	0.120	0.0043	105.0	6.75	1.24	0.05	0.027
Mono-Hex	1.000	--	0.127	0.0306	406.3	40.70	1.65	0.13	0.023
Di-Hex	1.000	--	0.012	0.0005	41.9	3.57	1.97	0.10	0.026
Tri-Quad	1.000	--	0.012	0.00005	34.1	1.49	1.65	0.02	0.044
Mono+S	1.000	--	0.027	0.0006	209.0	16.41	1.77	0.11	0.045
0T-4U	1.000	--	0.015	0.0005	98.8	11.22	2.32	0.17	0.0071
0T-8U	1.000	--	0.013	0.0006	58.4	10.77	2.15	0.24	0.010
0T-12U	1.000	--	0.019	0.0002	84.4	10.39	2.16	0.26	0.016
4T-8U	1.000	--	0.016	0.0009	83.0	6.20	2.21	0.32	0.0071
8T-4U	1.000	--	0.015	0.0007	101.8	3.45	2.31	0.13	0.0064
3:1 Di-Hex/0T-8U	1.000	--	0.067	0.023	257.8	42.09	1.47	0.19	0.020
6:1 Di-Hex/0T-8U	1.000	--	0.012	0.0002	41.9	7.28	1.92	0.14	0.016
10:1 Di-Hex/0T-8U	1.000	--	0.013	0.0002	15.9	1.20	2.20	0.18	0.017
20:1 Di-Hex/0T-8U	1.000	--	0.017	0.0003	6.9	0.64	1.90	0.12	0.022
0:1 BP:QD	1.000	--	0.012	0.0005	41.9	3.57	1.97	0.10	0.026
10:1 BP:QD	1.000	--	0.012	0.0002	20.1	0.89	1.94	0.08	0.015
20:1 BP:QD	1.000	--	0.012	0.0003	13.2	1.27	2.03	0.17	0.022
40:1 BP:QD	1.000	--	0.012	0.0002	8.6	0.45	1.98	0.05	0.036
Tabletop Assay	1.000	--	0.411	0.0162	17.2	1.52	1.95	0.22	0.011
6:1 Di-Hex/0T-8U**	1.000	--	-0.008	0.0128	14.4	0.67	1.61	0.11	0.032
40:1 BP:QD**	1.000	--	0.026	0.0017	4.4	0.11	1.83	0.08	0.017
40:1 Mono-Hex**	1.000	--	-0.068	0.0530	36.6	3.15	1.42	0.08	0.016
20:1 Tri-Quad**	1.000	--	0.030	0.0009	3.8	0.08	2.18	0.06	0.011
6:1 0T-12U**	1.000	--	0.083	0.0014	14.1	0.63	1.98	0.16	0.014

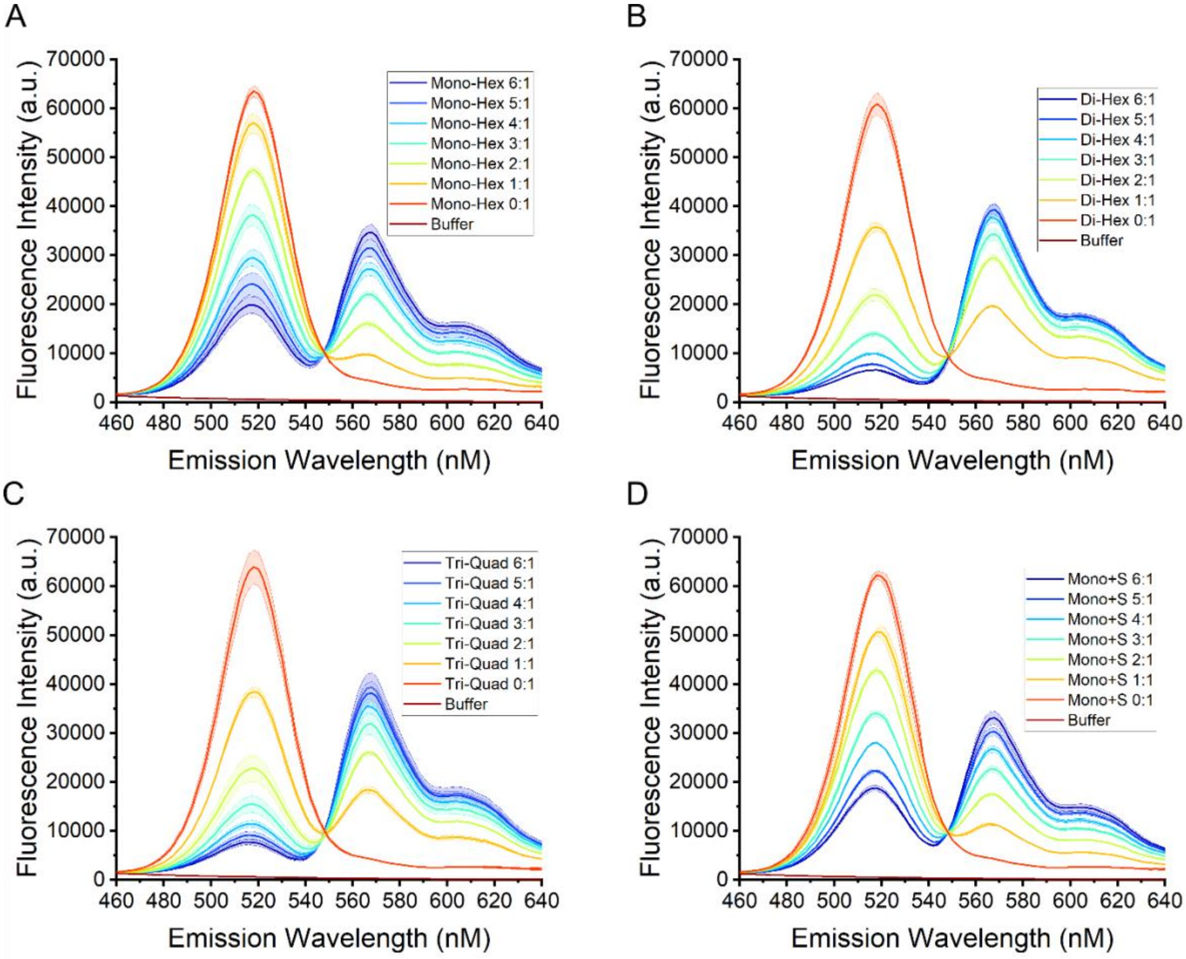

Figure S1 – Fluorescence spectra of QD molecular beacons assembled at varying acceptor to donor (A/D) molar ratios when excited at 320 nm. Four different peptide-PNA were used to assemble the molecular beacons, and the peptide-PNA used is indicated in the figure legends, namely (A) mono-hex histag, (B) di-hex histag, (C) tri-quad histag, and (D) mono+S histag. QD concentration was held constant at 100 nM, and peptide-PNA/hairpin-dye complexes were varied from 0 to 600 nM.

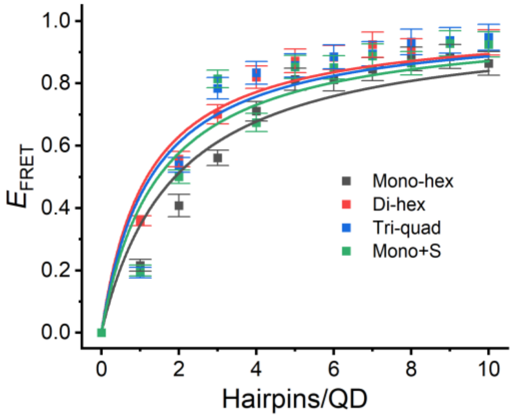

Figure S2 – FRET curves determined from the fluorescence spectra in Fig. S1 for the four peptide-PNA tested. The FRET efficiency (E_{FRET}) was determined using the relative change in QD fluorescence from the control with no acceptor (0:1 samples in Fig. S1).

Donor-acceptor distances (r_{DA}) reported in the main text were based on the centrosymmetric FRET assumptions of a QD donor and multiple organic fluorophore acceptors. We further assumed assembly at the tested concentrations to be approximately complete, with all acceptors assembled to donors. The Förster radius, R_0, was determined previously to be 5.5 nm for QD525 and Cy3.[1] The fitting equation is thus:

$$E_{FRET} = \frac{x * (R_0/r_{DA})^6}{1 + x * (R_0/r_{DA})^6} \tag{3}$$

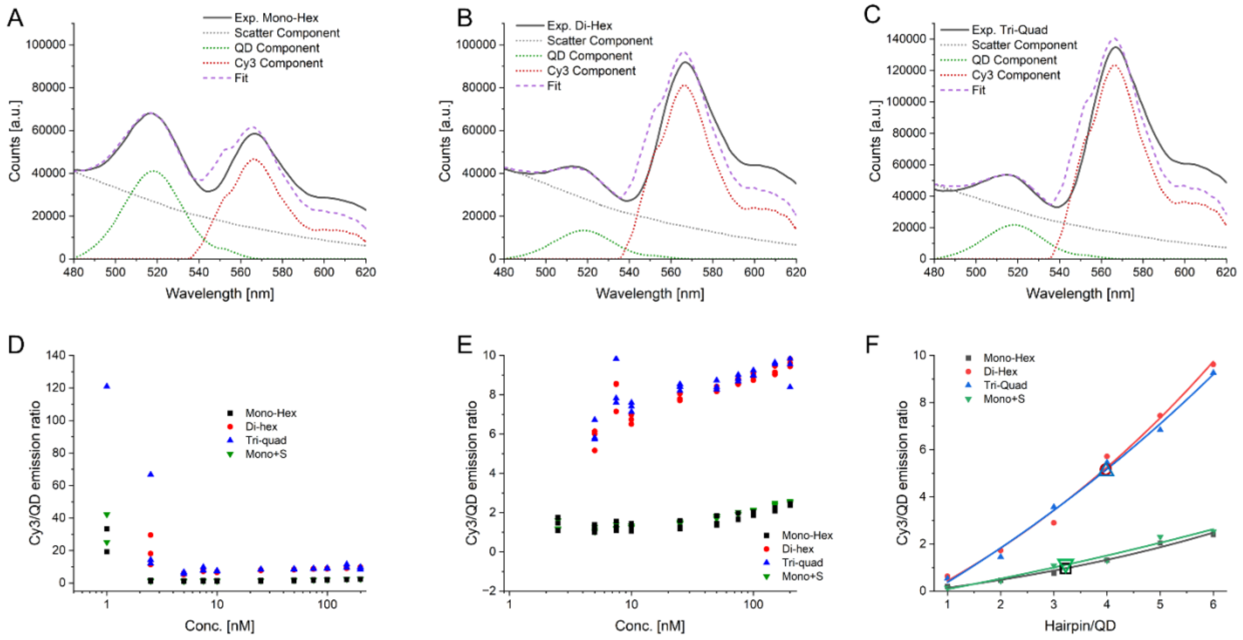

Figure S3 – Dilution assays to determine his-tag binding efficiency. A-C) Spectral decomposition using QD, Cy3, and scattering component of the fluorescence spectra of a 2 nM solution of QD with 6 acceptors bound through varying his-tags. A) Mono-hex. B) Di-Hex. C) Tri-Quad. D) Cy3/QD emission ratio based off of the deconstructed spectra as a function of sample concentration. Data obtained in triplicate (all data shown as individual data points). E) Zoomed in data from previous figure. F) Calibration curve of Cy3/QD emission ratio as a function of the number of acceptors. The empty points correlate to the measured Cy3/QD emission at 2.5 nM.

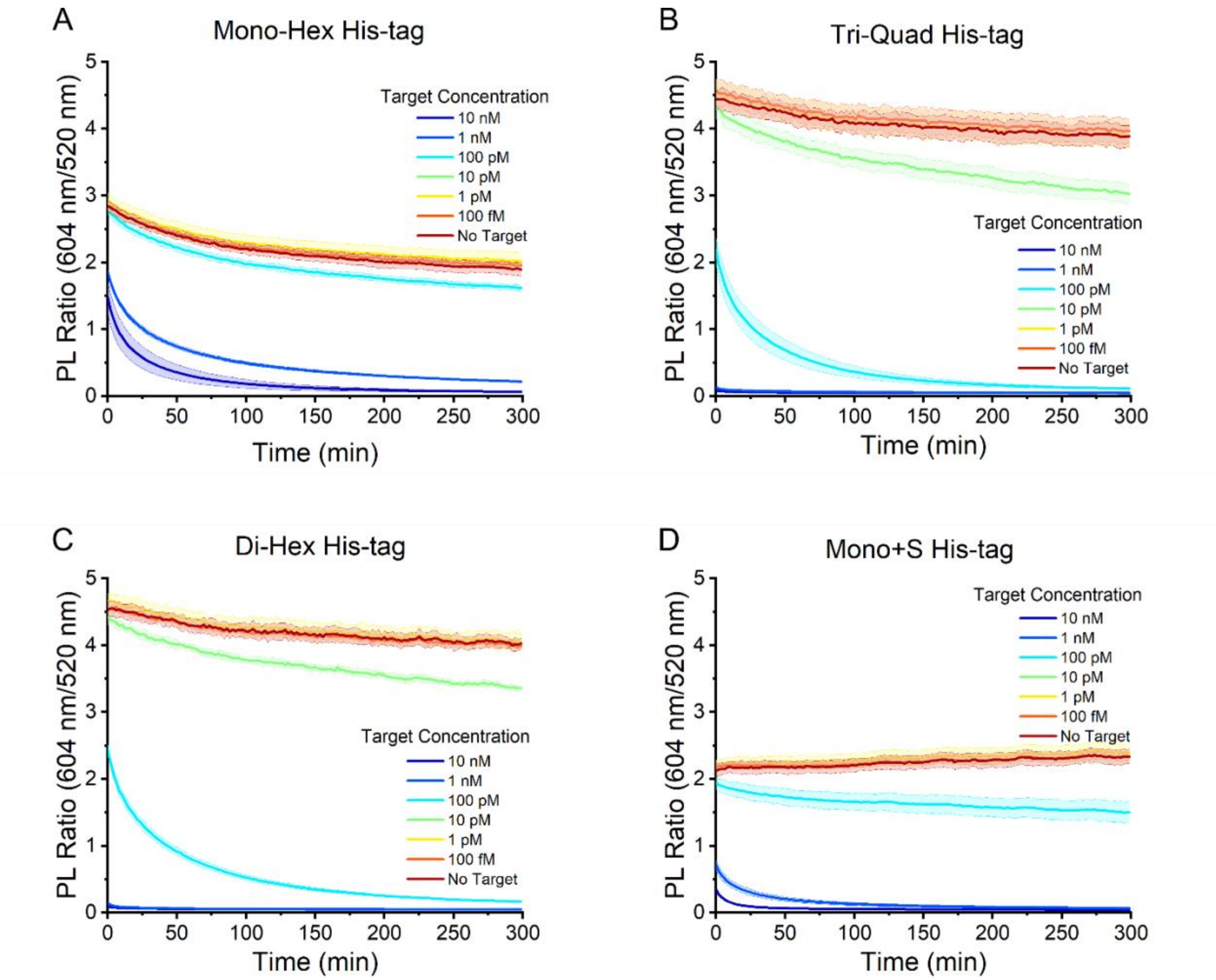

Figure S4 – CRISPR-Cas target dilution assays to compare the effects of histag design for target concentrations from 10 nM down to 100 fM. All his-tag variations were run at 6:1 acceptor to donor molar ratio. Photoluminescence (PL) ratio time traces are shown for QD-MBs assembled using (A) mono-hex his-tag, (B) tri-quad his-tag, (C) di-hex his-tag, and D) mono+S his-tag.

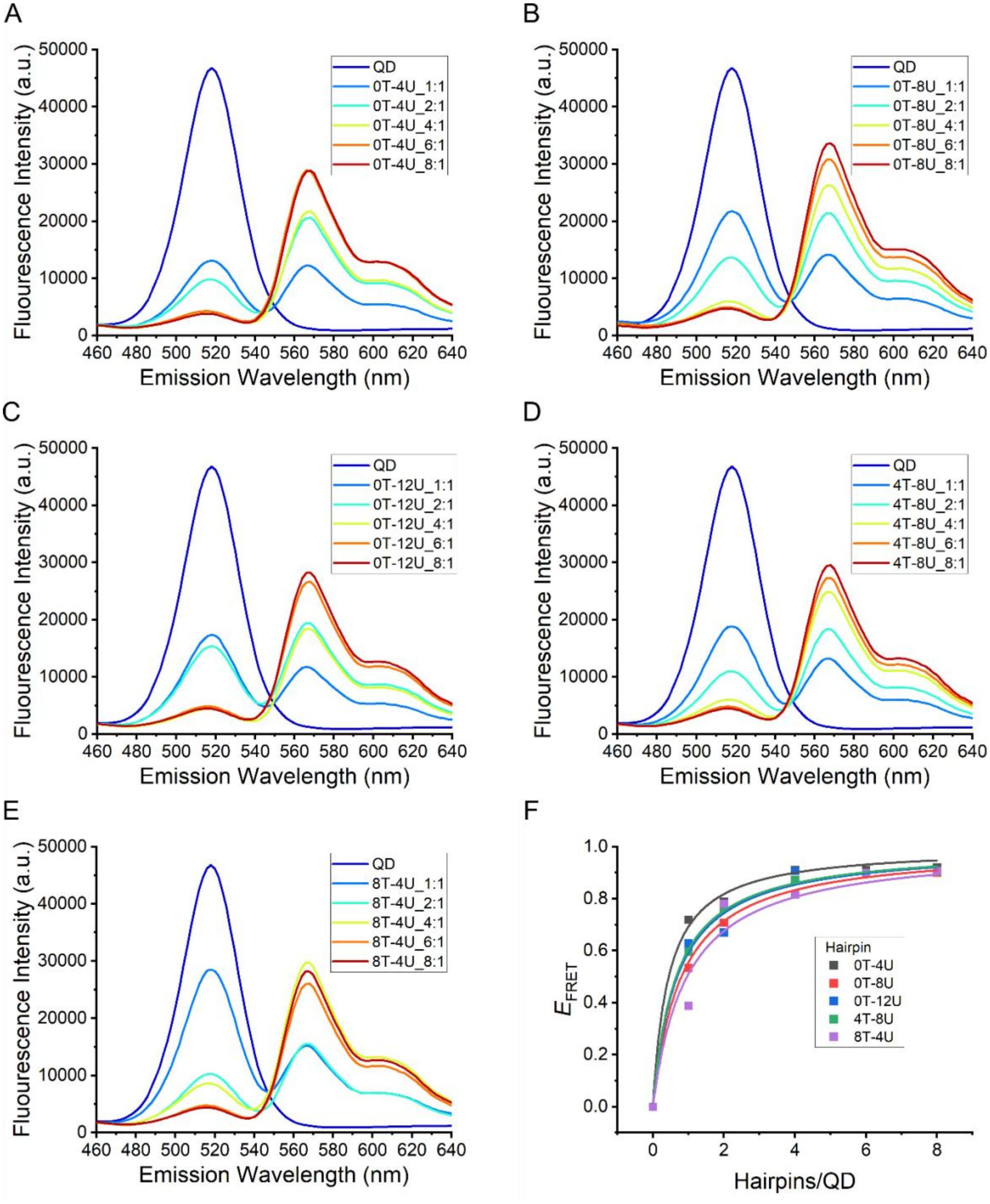

Figure S5 – Fluorescence spectra of QD molecular beacons assembled with five different chimeric RNA hairpins, namely (A) 0T-4U, (B) 0T-8U, (C) 0T-12U, (D) 4T-8U, and (E) 8T-4U. All QD molecular beacons were assembled using the di-hex his-tag. F) FRET efficiency curves for hairpin to quantum dot ratios of 1:1, 2:1, 4:1, 6:1 and 8:1.

To assess whether the introduction of surfactants into the CRISPR-Cas QD-MB assays would improve assay performance by reducing non-specific binding of DNA and proteins to surfaces, CRISPR-Cas QD-MB assays were performed with surfactants Triton x-100 and Tween-20 at 0.1% and compared against a control lacking surfactant. The results (Fig. S6) showed no significant differences between the control without surfactant and the experimental runs with surfactants. As stated in the main text, surfactants were thus not included in further experiments.

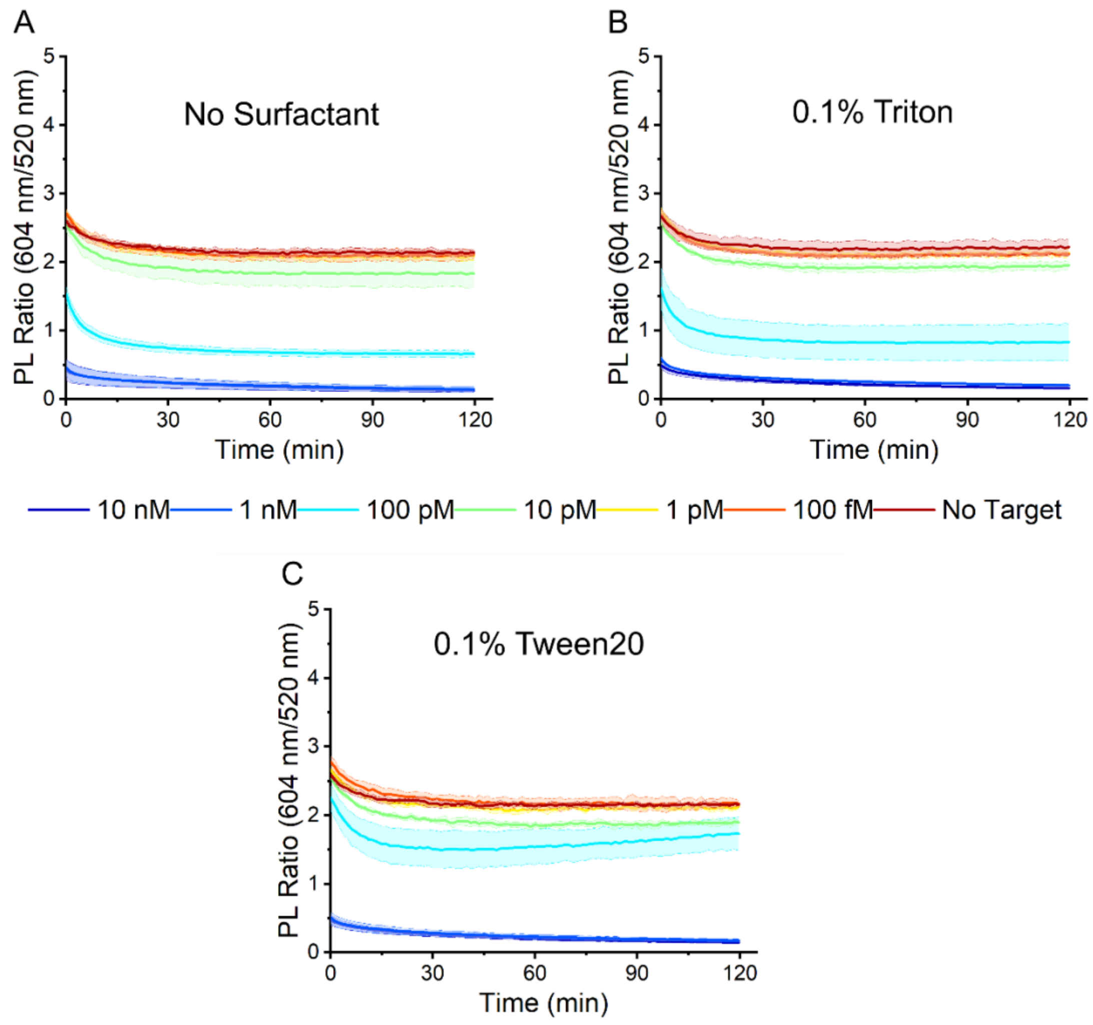

Figure S6 – CRISPR-Cas target dilution assays performed with A) no surfactant, B) 0.1% of Triton x-100, and C) 0.1% Tween-20. The surfactants were added to the standard Cas assay running buffer composed of 50 mM Tris-HCl, 10 mM $MgCl_2$, and 100 mM NaCl. QD-MBs were constructed with di-hex his-tags and 0T-8U hairpins at an acceptor to donor molar ratio of 6:1 with 100 nM QD.

To determine if CRISPR-Cas assays were being hindered by adsorption of target strand to surfaces during assay preparation, a poly-T DNA strand comprised of 30 nucleotides of thymine was added at a 1 uM concentration to the working solution of target strands before diluting and mixing into microplate wells for assay initialization. The results (Fig. S7) suggest that poly-T may benefit detection at low target concentrations, though the results were not statistically significant within the standard 60 min assay window due to increased deviation between replicate samples.

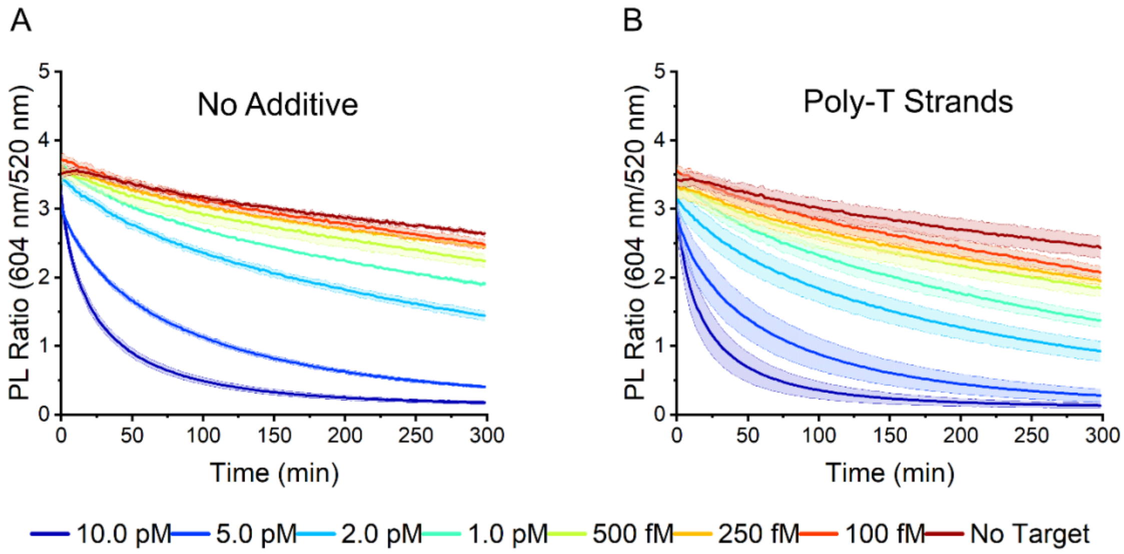

Figure S7 – CRISPR-Cas target dilution assays performed with (A) no non-target DNA and (B) additional poly-T DNA strands for surface passivation. For poly-T containing samples, 1 uM of poly-T strands were mixed into the vial of target strands prior to pipetting. QD molecular beacons were assembled with tri-quad his-tags and 4T-8U hairpin at an acceptor to donor molar ratio of 20:1 and 100 nM QD; assembly was performed at 25 ºC.

During the last phase of the experiments, the original QD stock was expended. To test the replicability of the results with QDs possessing varied surface ligand coverage (assumed to be an effect of aging as observed by changes in QD solubility over time), a fresh resuspension and ligand exchange of QDs from the same QD batch was mixed. The original QD mixture and the new QD mixture were both assembled with tri-quad his-tags and 0T-12U hairpins at 6:1 A/D ratios and run in CRISPR-Cas target dilution assays for comparison. Fig. S8 shows the PL ratio time traces for old and new QDs, in which it was observed that the old QDs had greater surface coverage of the his-tag/hairpin complex as indicated by higher initial PL ratios. This observation was in line with the assumption that new QDs would possess higher surface ligand density and thus less surface area available for his-tag binding. Despite the increased binding, the old QDs performed worse overall than the new QDs, further supporting the conclusions from the main text that blocking peptides and excess his-tag/hairpin complexes improved QD-MB performance through increased surface passivation of QDs.

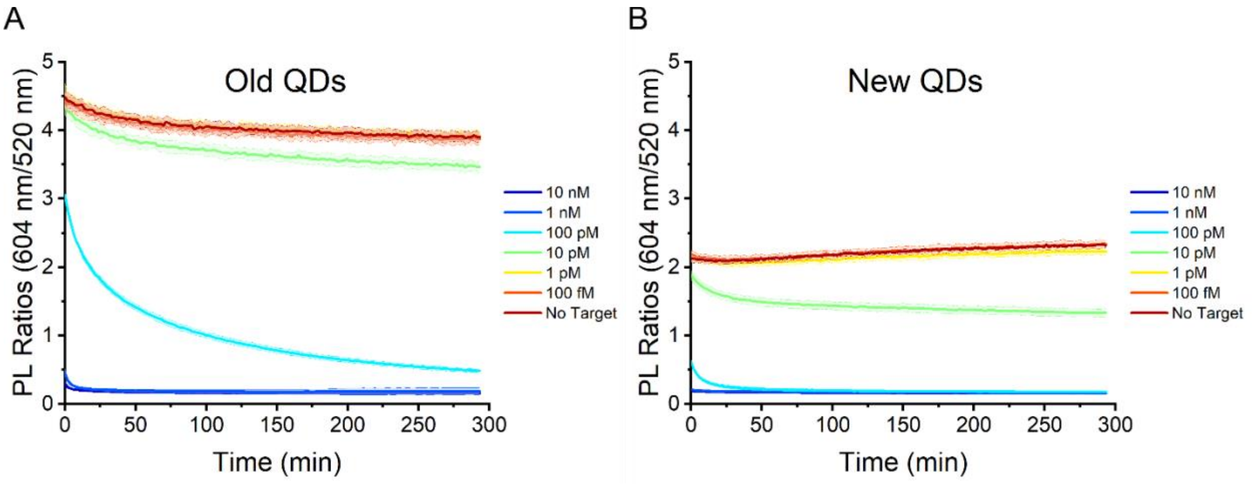

Figure S8 – CRISPR-Cas target dilution assays performed using (A) old and (B) new quantum dots for preparation of the QD-MB. QD-MBs were assembled using the tri-quad his-tag and 0T-12U hairpin at A/D ratios of 6:1. Old QDs showed PL ratios stabilizing near 4, while new QDs had initial PL ratios of around 2.2 and continued to increase throughout the experiment. The slow but continuous increase in the PL ratio for new QDs suggests that QD-MB assembly may not have been complete prior to the start of the experiment.

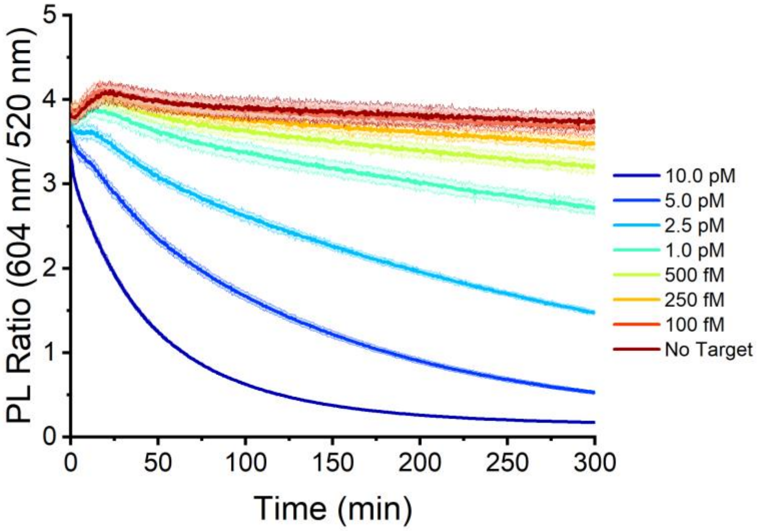

Figure S9 – CRISPR-Cas target dilution assay performed under the optimized conditions identified in the main text. QD-MBs were constructed with di-hex his-tags and 0T-8U hairpins assembled in significant excess to the QD (40:1). Target concentrations are indicated in the figure legend.

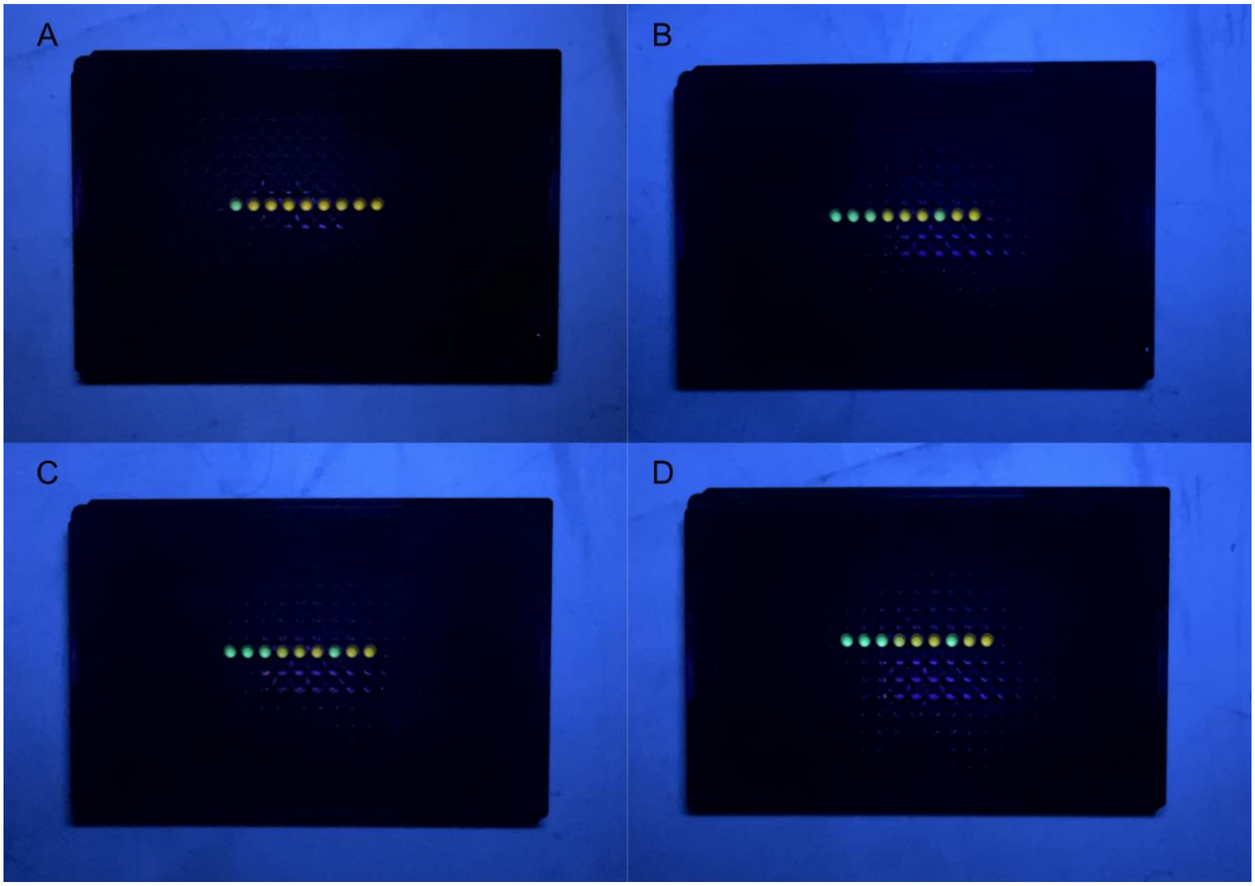

Figure S10 – Raw images of samples in a 384 well, low volume microplate acquired using the countertop UV lamp setup shown in Fig. 5 of the main text. The setup consists of a 365 nm UV lamp, cardboard box, and an iPhone 13 Pro using the app ProCam – Pro Camera to manually set ISO, shutter speed, and f-stop (*f*) and deactivating automated image adjustments. Images were acquired at (A) 0 min, (B) 30 min, (C) 60 min, and (D) 120 min, with time 0 being acquisition of the first image. Approximately 1 min passed between target addition and the first image acquisition. Images were acquired with ISO 8000, wide camera – 26 mm *f* 1.5, and 1/15 s exposure.

References

1 Green, C. M. *et al.* Quantum dot-based molecular beacons for quantitative detection of nucleic acids with CRISPR/Cas (N) nucleases. *ACS Nano* **16**, 20693-20704 (2022).
